# *Cyclopia* Extracts Act as ERα Antagonists and ERβ Agonists, *In Vitro* and *In Vivo*

**DOI:** 10.1371/journal.pone.0079223

**Published:** 2013-11-04

**Authors:** Koch Visser, Morné Mortimer, Ann Louw

**Affiliations:** Department of Biochemistry, University of Stellenbosch, Matieland, Stellenbosch, Republic of South Africa; University of Wisconsin - Madison, United States of America

## Abstract

Hormone replacement therapy associated risks, and the concomitant reluctance of usage, has instigated the search for new generations of estrogen analogues that would maintain estrogen benefits without associated risks. Furthermore, if these analogues display chemo-preventative properties in breast and endometrial tissues it would be of great value. Both the selective estrogen receptor modulators as well as the selective estrogen receptor subtype modulators have been proposed as estrogen analogues with improved risk profiles. Phytoestrogen containing extracts of *Cyclopia*, an indigenous South African fynbos plant used to prepare Honeybush tea may serve as a source of new estrogen analogues. In this study three extracts, P104, SM6Met, and cup-of-tea, from two species of *Cyclopia*, *C. genistoides* and *C. subternata*, were evaluated for ER subtype specific agonism and antagonism both in transactivation and transrepression. For transactivation, the *Cyclopia* extracts displayed ERα antagonism and ERβ agonism when ER subtypes were expressed separately, however, when co-expressed only agonism was uniformly observed. In contrast, for transrepression, this uniform behavior was lost, with some extracts (P104) displaying uniform agonism, while others (SM6Met) displayed antagonism when subtypes were expressed separately and agonism when co-expressed. In addition, breast cancer cell proliferation assays indicate that extracts antagonize cell proliferation in the presence of estrogen at lower concentrations than that required for proliferation. Furthermore, lack of uterine growth and delayed vaginal opening in an immature rat uterotrophic model validates the ERα antagonism of extracts observed *in vitro* and supports the potential of the *Cyclopia* extracts as a source of estrogen analogues with a reduced risk profile.

## Introduction

Hormone replacement therapy (HRT), estrogens alone or in combination with progestins, is traditionally prescribed to women undergoing menopausal transition to alleviate symptoms associated with menopause [1], such as hot flashes, night sweats, sleeping problems, vaginal dryness, and osteoporosis [2-4]. However, a number of side effects have been associated with the use of HRT, for example, an increased occurrence of breast cancer [5,6], vaginal bleeding [7], and heart disease or strokes [6,8]. These side effects have led to reluctance among concerned consumers to use HRT and instigated a search for new estrogen analogues with an improved risk profile. Furthermore, it would be of great value if these analogues should also display chemo-preventative properties in breast tissue [9,10].

Estrogens elicit their biological effects by binding to transcription factors called estrogen receptors (ERs) in the target organ/tissue (uterus, ovary, vagina, liver, bone, and breast) [11-13]. The ER exists as two subtypes, namely ERα and ERβ [14]. Current estrogens in HRT activate both subtypes of ER in all tissues [14-19]. This attribute is beneficial in bone [18,20,21] and for hot flashes [18,21], but detrimental in the breast [6,21,22] and uterus [21,23] as it increases the risk of tumorigenesis. In contrast, the selective estrogen receptor modulators (SERMs), although not ER subtype specific [24,25], act as agonists in certain tissues, such as bone [26-28], and as antagonists in others, such as breast [9,10,29]. Although, the well-known SERMs, raloxifene and tamoxifen [30], have been shown to decrease the risk of breast cancer [18,31,32] and increase bone mineral density [26-28,33], they have also been linked to an increased risk of venous thromboembolism and occurrence of hot flashes, and can stimulate endometrial growth [28,34-36]. SERMs are thus not considered as suitable alternatives for HRT. 

Physiologically, while ERα is associated with the promotion of cell proliferation that contributes to the occurrence of breast and endometrial cancer, several studies have shown that ERβ inhibits ERα-dependent cell proliferation and could prevent cancer development [15,22,37-43]. 17β-estradiol (E_2_) has similar binding affinities for the two ER subtypes [44], and the subtypes stimulate the transcription of both common and distinct subsets of E_2_ target genes [13,17,39,45]. However, in many cases the degree of activation via ERβ is lower [44], despite the high ligand independent transcriptional activity of this subtype [46,47]. In light of the above, it has been suggested that the development of ER subtype specific ligands may herald the arrival of a new generation of estrogen analogues that may present a novel treatment for post-menopausal symptoms, which in addition, may prevent or decrease the occurrence of breast cancer [44,48,49]. An ideal or “designer” estrogen analogue or selective estrogen receptor subtype modulator (SERSM) has been postulated that would have the following attributes: act as an ERα selective antagonist [50], down-regulate ERα protein levels [50,51], selectively activate ERβ transcriptional pathways [15,19,24,43], and display anti-inflammatory properties by inhibiting transcription of pro-inflammatory genes to prevent the occurrence of post-menopausal osteoporosis [15,52]. Current examples of subtype specific ligands are, methyl-piperidino-pyrazole (MPP) (ERα antagonist) [53,54], diarylpropionitrile (DPN) (ERβ agonist) [55], ERB-041 (ERβ agonist) [56,57], liqueritigenin (ERβ agonist) [19], isolated from the plant extract MF101 (ERβ agonist) [24]. Phytoestrogens have been referred to as natural SERMs and can be both estrogenic as well as antiestrogenic [58-60]. Furthermore, although evidence in the literature shows that phytoestrogens can bind to both ER subtypes, they generally have a higher affinity for the ERβ subtype [61-63] as well as a higher transcriptional potency and efficacy via ERβ [63]. Despite conflicting evidence regarding doses of phytoestrogens and breast cancer risk [64,65], generally, findings have pointed the search in the direction of phytoestrogens and focused attention on phytoestrogen rich food sources as a possible source of the ideal SERSM.

One such source may be *Cyclopia* (family: Fabaceae), an indigenous fynbos plant from the Western Cape province of South Africa [66,67]. Traditionally, the “fermented” (oxidized) form of *Cyclopia*, has been consumed as a fragrant, caffeine free honeybush tea beverage with the “unfermented” form being introduced to the commercial market more recently [63,67,68]. Studies that investigated the chemical composition of *Cyclopia* have shown that phenolic compounds with estrogenic activity, for example luteolin, eriodictyol, naringenin, and formononetin, are present in various species of *Cyclopia* [63,68-72]. Furthermore, although dried methanol extracts (DMEs) from plant material of two species of *Cyclopia*, *C. genistoides* and *C. subternata*, have been shown to bind to the ERs and are able to transactivate an ERE-containing promoter reporter construct [62,63,68], only the extract from *C. genistoides* was investigated for ER subtype specificity and found to transactivate only through ERβ, despite binding to both subtypes [62,63]. In addition, studies by Verhoog et al. [63] and Mfenyana et al. [68] showed that although extracts of *Cyclopia* are able to induce proliferation of the ERα and ERβ positive MCF-7 BUS cells, they antagonise E_2_ induced cell proliferation.

The current study was prompted by the findings of Verhoog et al. [62,63] that the *Cyclopia* extract, P104, although binding to both receptors and with a much higher affinity for ERα, was able to activate an ERE-containing promoter reporter construct only via ERβ. As the possibility of ERα antagonism by *Cyclopia* extracts had not been addressed in previous studies it appeared essential to evaluate ERα antagonism while also re-evaluating ERβ agonism. The combination of ERα antagonism and ERβ agonism may be especially relevant for the chemoprevention of breast cancer as ER antagonism serves as the basis of current chemo-preventative agents [29,31,32,73,74], while ERβ specific agonists have recently been identified as having potential for the chemoprevention of breast cancer [19,22]. In addition, this combination might be advantageous for the treatment of menopausal symptoms as an ERβ agonist has been shown to alleviate both hot flashes and the surge of inflammation related diseases during menopause [24,52], while an ERα antagonist would not result in hyperplasia of the uterus, commonly associated with ERα agonists [15,52]. Thus, in this study, we evaluate the potential of several extracts of *Cyclopia* to act as ERα antagonists and ERβ agonists and demonstrate that all extracts display ERα antagonism, while two also display ERβ agonism. In addition, all extracts antagonise E_2_-induced MCF-7BUS cell proliferation, one extract displays anti-inflammatory activity, and the two tested extracts do not stimulate uterine growth. These results suggest that the *Cyclopia* extracts, which display ERα antagonism and ERβ agonism, have positive attributes that could possibly be further exploited for the development of safer drugs for the treatment or prevention of osteoporosis or pre-menopausal symptoms.

## Material and Methods

### Ethics statement

Animal care and experimental procedures were conducted with strict adherence to the accepted standards for the use of animals in research and teaching as reflected in the South African National Standards 10386: 2008. Stellenbosch University ethics committee approved this study (ethical approval reference: 11NB_LOU01).

### Test Compounds

17β-Estradiol (E_2_), genistein, luteolin, enterodiol, phorbol 12-myristate 13-acetate (PMA) and fulvestrant (ICI 182,780) were obtained from Sigma-Aldrich^®^, South Africa, and coumestrol was obtained from Fluka™ Analytical, Sigma-Aldrich^®^, South Africa. The *Cyclopia* extracts used for *in vitro* studies, P104 [62], SM6Met [68] and cup-of-tea [68], were previously prepared, while for *in vivo* studies new SM6Met and cup-of-tea extracts were prepared as previously described [68]. E_2_, genistein, luteolin, enterodiol, coumestrol and *Cyclopia* extract stock solutions were prepared in dimethylsulfoxide (DMSO).

### High-performance liquid chromatography (HPLC) analysis of C. *subternata* extracts

The newly prepared SM6Met and cup-of-tea extracts were analyzed using HPLC. Extracts and stock solutions of standards were prepared in DMSO and aliquots frozen at -20°C until needed for analysis. For experimental analysis ascorbic acid was added to defrosted standards and extracts to a final concentration of 9.8 mg/ml. The mixtures were then filtered using Millex-HV syringe filters (Millipore) with a 0.22 µm pore size. 

Analyses were performed on an Agilent 1200 HPLC consisting of an in line degasser, diode-array detection (DAD), column oven, autosampler and quaternary pump, controlled by Chemstation software (Agilent Technologies, Santa Clara, CA). The HPLC method previously described by De Beer et al. [75] was used to quantify the major phenolic compounds in *C. subternata* extracts: *A Gemini*-NX C18 (150 × 4.6 mm; 3 μm; 110 Å) column was used in conjunction with 2% acetic acid (A) and acetonitrile (B) as mobile phases. Injection volumes ranged from 10-20 µl for standards and 5-50 µl for the extracts. Separation was performed at a flow rate of 1 ml/min with the following mobile phase gradient: 0-2 min (8% B), 2-27 min (8-38% B), 27-28 min (38-50% B), 28-29 min (50% B), 29-30 min (50-8% B), 30-40 min (8% B); at a temperature of 30°C.

The dihyrochalcones, flavanones and benzophenones were quantified at 288 nm, whereas the xanthones, flavones and phenolic acids were quantified at 320 nm. A calibration curve consisting of seven points was set up for all the available standards (mangiferin (Sigma-Aldrich®, South Africa), isomangiferin (Chemos GmbH, Germany), luteolin (Extrasynthese, France), eriocitrin (Extrasynthese, France), hesperidin (Sigma-Aldrich®, South Africa), protocatechuic acid (Fluka™ Analytical, Sigma-Aldrich^®^, South Africa)) and also standards needed to calculate equivalent values (aspalathin (kind gift from Prof. Gelderblom, PROMEC unit, Medical Research Council, Tygerberg, South Africa), apigenin (Fluka™ Analytical, Sigma-Aldrich®, South Africa), and nothofagin (kind gift from Prof. Gelderblom, PROMEC unit, Medical Research Council, Tygerberg, South Africa)). Iriflophenone-3-*C-β-*glucoside and iriflophenone-di-*O*,*C*-hexoside was quantified using iriflophenone-3-*C*-glucoside isolated from *C. genistoides* (personal communication from Dr. D. de Beer). Scolymoside and vicenin-2 were expressed as luteolin and apigenin equivalents, respectively, as no authentic reference standards were available for these compounds. Also phloretin-3',5'-di-C-glucoside was expressed in terms of nothofagin (3-hydroxyphloretin-3'-*C*-glucoside) equivalents.

### Cell Culture

COS-1, African green monkey kidney fibroblast cells (ATCC, United States of America), and MCF-7BUS human breast cancer cells [76] (a kind gift from A. Soto, Tufts University, Boston, Massachusetts, United States of America) were maintained in high glucose (4.5 g/L) Dulbecco’s modified eagle’s medium (DMEM) (Sigma-Aldrich^®^) supplemented with 10% FCS (Highveld Biologicals, South Africa), 100 IU/ml penicillin and 100 µg/ml streptomycin (Gibco, Invitrogen™, South Africa), 2mM glutamine (Merck), 44mM sodium-bicarbonate (Gibco), 1mM sodiumpyruvate (Gibco, Invitrogen Corporation), and 0.1mM non-essential amino acids (Gibco). All cells were maintained in a humidified cell incubator, set at 97% relative humidity and 5% CO_2_ at 37°C. For the cell proliferation assays (MTT assay) MCF-7BUS cells were withdrawn from 100 IU/ml penicillin and 100µg/ml streptomycin for seven days prior to use.

### MTT assay

On day one MCF-7BUS cells were seeded into 96-well tissue culture plates at a concentration of 2500 cells/well and allowed 24 hours to settle. The next day cells were washed with 200 µl/well pre-warmed PBS and the medium was changed to DMEM without phenol red supplemented with 5% charcoal treated FCS (Highveld Biologicals, South Africa) and incubated for 24 hours. After incubation the cells were treated for 48 hours with increasing concentrations test compounds and *Cyclopia* extracts in the presence or absence of 10^-9^M E_2_ where after the colorimetric MTT (3-(4,5- dimethylthiazolyl-2)-2,5-diphenyltetrazolium bromide) assay, adapted from Verhoog et al. [63] and Mfenyana et al. [68], was performed. Briefly, the MTT assay entails that 4 hours before the end of the incubation period the assay medium is changed to 150 µl DMEM without phenol red, but supplemented with 5% charcoal stripped FCS, and 50 µL of MTT (methylthiazolyldiphenyl-tetrazolium ) (Sigma-Aldrich^®^) solution (5 mg/ml) is added to each well. Cells are then incubated for four hours at 37°C, the medium removed, and 200 µL of solubilisation solution (DMSO) added to each well. The plate is then covered with foil, shaken at room temperature for 5 min, and the absorbance read at 550 nm on a BioTek^®^ PowerWave 340 spectrophotometer. All assays included a negative solvent control, which consisted of 0.1% (v/v) DMSO only. Results are expressed as fold induction relative to solvent.

### Promoter reporter studies

MCF-7BUS and COS-1 cells were seeded in sterile 10 cm tissue culture plates at a concentration of 2 x 10^6^ cells/plate and allowed 24 hours to settle. On day two the cells were rinsed once with sterile phosphate buffered saline (PBS) (pre-warmed to 37°C), medium changed to DMEM without phenol red supplemented with 10% charcoal treated FCS and 1% penicillin and streptomycin mixture, and cells were transfected. 

#### Plasmids

Human (h) ERα (pSG5-hERα [77]) and ERβ (pSG5-hERβ [78]) expression plasmids were kind gifts from F. Gannon (European Molecular Biology Laboratory, Heidelberg, Germany). The ERE-containing promoter reporter construct (ERE.vit2.luc) was a kind gift from K. Korach, National Institute of Environmental Health Science, U.S. [79] and the NFκB-containing promoter reporter construct (p(IL6κB)350hu.IL6Pluc+ [80]) was a kind gift from G. Haegeman, University of Ghent, Ghent, Belgium. pGL2-Basic (Promega Corporation, Madison, Wisconsin, U.S.A.) was used as an empty vector.

#### Transactivation

To test transactivation through ERα COS-1 cells were transfected with 150 ng hERα and 6000 ng of an ERE-containing promoter reporter construct. To test transactivation through ERβ COS-1 cells were transfected with 150 ng hERβ, 3000 ng of an ERE-containing promoter reporter construct, and 3000 ng empty vector. MCF-7 BUS cells (which contain endogenous hERα and hERβ) were transfected with 3000 ng of an ERE-containing promoter reporter construct and 3000 ng empty vector. The amount of promoter reporter construct for each test model that was selected was determined by the highest E_2_ induction achieved (Figure S1).

#### Transrepression

To test transrepression through ERα COS-1 cells were transfected with 150 ng hERα, 1500 ng of an NFκB-containing promoter reporter construct and 4500 ng empty vector. To test transrepression through ERβ COS-1 cells were transfected with 150 ng hERβ, 4500 ng of an NFκB-containing promoter reporter construct and 1500 ng empty vector. MCF-7BUS cells (which contain endogenous hERα and hERβ) were transfected with 6000 ng of an NFκB-containing promoter reporter construct. The amount of promoter reporter construct for each test model that was selected was determined by the most effective E_2_ repression of PMA induction achieved (Figure S2)

All transfections were performed using FuGENE^TM^ 6 transfection reagent (Roche Applied Science, South Africa) as described by the manufacturer. Cells were left for 24 hours, replated in sterile 24-well tissue culture plates at a concentration of 5 x 10^4^ cells/well and allowed 24 hours to settle. Cells were treated for 24 hours with test compounds and *Cyclopia* extracts and lysed overnight with 50 µl lysis buffer [0.2% (vol/vol) Triton, 10% (vol/vol) glycerol, 2.8% (vol/vol) Tris-phosphate-EDTA, and 1.44 mM EDTA] per well at -20 °C. Luciferase activity was determined using the luciferase assay kit (Promega Corporation, Anatech, South Africa) according to the manufacturer’s instructions and normalized for protein content (Bradford assay [81]). Results are expressed as fold induction relative to solvent.

### Western Blot

Cell lysates from COS-1 cells transfected with either ERα (150 ng hERα/10 cm plate) or ERβ (150 ng hERβ/10 cm plate) and MCF-7BUS cells were prepared by adding lysis buffer A (10mM Hepes pH 7.5 (Gibco, Invitrogen Corporation), 1.5mM MgCl_2_, 10mM KCl, 0.1% NP-40 (Roche Applied Science) and Complete Mini protease inhibitor cocktail (Roche Applied Science), shaking on ice for 15 min and frozen overnight at -20°C.

On thawing, lysate were transferred to 1.5ml Eppendorf tubes on ice, centrifuged for 10 min at 12 000 x g at 4°C and the cleared lysate was transferred to 1.5ml Eppendorf tubes on ice, alliquoted and stored at -20°C until assayed. Lysates (20µl) were separated on a 10% SDS-PAGE gel. Following electrophoresis, proteins were electro-blotted and transferred to a Hybond-ECL nitrocellulose membrane (Amersham Biosciences, South Africa), which was probed for ERα (diluted 1:500), ERβ (1:250) and GAPDH (1:500). Proteins were visualized using HRP labeled anti-rabbit antibody for ERα (1:2500) and ERβ (1:1000), or HRP labeled anti-mouse antibody for GAPDH (1:5000), and ECL Western blotting detection reagents (Pierce^®^, Thermo Fisher Scientific Inc., U.S.A.) and medical x-ray film (Axim (PTY) LTD., South Africa). All antibodies, primary [ERα (HC-20), cat# sc-543, ERβ (H-150), cat# sc-8974, and GAPDH (0411), cat# sc-47724] and secondary (anti-rabbit, cat# sc-2005, and anti-mouse, cat# sc-2030), were purchased from Santa Cruz Biotechnology, Inc., U.S.A. 

### Animal care

Immature female Wistar rats were obtained from the Stellenbosch University, South Africa, breeding unit and were received as weanlings on postnatal day 18. The animals had free access to standard rat feed (Pure Harvest Rat Feed, Afresh Vention (PTY) Ltd, South Africa) and drinking water. The animals were housed in a 12 hour light-dark cycle at a constant temperature of 20 °C in EHRET individually ventilated cages (EHRET, Emmedingen, Germany). The animals were allowed at least 24 hours to acclimatize before the onset of experimental procedures. 

### Immature rat uterotrophic assay

The immature rat uterotrophic assay was performed according to methods previously described by Kanno et al. [82] and de Lima et al. [83]. Immature Wistar rats (21 days) were randomly assigned to groups (n=10) and treated daily with E_2_, genistein, *Cyclopia* extracts, or vehicle control (sterile PBS) by oral gavage for three consecutive days. The dose volume was 200 μl/day. The test compounds were suspended in sterile PBS and the solution was kept homogenous by stirring before administration. General health, vaginal opening, and body weight was monitored daily and recorded. On day four, approximately 24 hours after last dose, animals were weighed and sacrificed by administration of a high dose of Isoflurane (2-chloro-2-(difluoromethoxy)-1,1 1-trifluoro-ethane), (Safeline pharamceuticals Pty (Ltd)). Livers were removed and weighed. Uteri were removed, cleaned of excess fat, photographed, weighed, pierced to remove luminal fluids, and blotted uterine weights were obtained immediately. 

### Evaluation/Monitoring of vaginal opening of Wistar rats for extended period

Immature Wistar rats (21 days) were randomly assigned to groups (n=10) and treated daily with E_2_, *Cyclopia* extracts, or vehicle control (sterile PBS) by oral gavage for 30 consecutive days. The dose volume had to be increased gradually from 200 μl/day to 400 μl/day as animals increased in body weight. The test compounds were suspended in sterile PBS and the solution was kept homogenous by stirring before administration. General health, vaginal opening, and body weight was monitored daily and recorded. On day 30 animals were weighed and sacrificed by administration of a high dose of Isoflurane.

### Data manipulation and statistical analysis

The GraphPad Prism® version 5.10 for Windows (GraphPad Software) was used for graphical representations and statistical analysis. One-way ANOVA and Dunnett’s post-test comparing all columns to the solvent control were used for statistical analysis and significance is displayed on the graphs. For all experiments the error bars represent the SEM of at least two independent experimental repeats, except for *in vivo* studies where the error bars represent the SEM of the number of animals used.

## Results

### HPLC analyses of extracts of *Cyclopia*

New SM6Met and cup-of-tea extracts were prepared from the same harvesting of *C. subternata* previously used to prepare these extracts [68]. HPLC analysis was performed on these newly prepared SM6Met and cup-of-tea extracts (Table 1). Prior HPLC results of previously prepared P104 [63] and SM6Met [68] extracts are also shown in Table 1. The results indicate the presence of the xanthones, mangiferin and isomangiferin, the flavones, scolymoside, luteolin, and vicenin-2, the flavanones, eriocitin and hesperidin, the dihydrochalcones, phloretin-3,5-di-C-glucoside and aspalathin, the benzophenones, iriflophenone-3-*C*-β-glucoside and iriflophenone-di-*O*,*C*-hexoside, and the phenolic carboxylic acid, protocatechuic acid. P104, a DME from *C. genistoides*, contained more mangiferin and isomangiferin than SM6Met, a DME from *C. subternata*, while, the cup-of-tea extract from the same species contained the least. Luteolin was present in all of the extracts, albeit at small amounts, with the P104 extract containing the largest amount, followed by the SM6Met extracts, and with the cup-of-tea extract containing the least. The luteolin rutinoside, scolymoside, was not evaluated in P104. The DMEs of *C. subternata* contained more scolymoside, eriocitrin, hesperidin, and phloretin-3,5-di-C-glucoside than the cup-of-tea extract. The newly prepared DME, SM6Met, contained higher amounts than the cup-of-tea extract of compounds not previously tested for, namely, iriflophenone-3-*C*-β-glucoside, iriflophenone-di-*O*,*C*-hexoside, aspalathin, vicenin-2, and protocatechuic acid. In general the DMEs contained higher concentrations of the polyphenols quantified (Table 1) than the water extract.

**Table 1:** Major polyphenols present in previously and newly prepared extracts of *Cyclopia* as determined by HPLC.

	Extract			
	Previously prepared		Newly prepared	
Polyphenol [% of dry extract (g/100g dry extract)]	P104 [63] *C.genistoides*	SM6Met [68] *C.subternata*	SM6Met *C.subternata*	Cup-of-tea *C.subternata*
Mangiferin	3.606	1.850	1.899	1.000
Isomangiferin	5.094	0.750	0.645	0.420
Luteolin	0.096	0.040	0.040	0.018
Scolymoside (luteolin-7-*O*-rutinoside)	nt^a^	1.820^*c*^	1.289	0.876
Vicenin-2 (apigenin-6,8-di-C-glucoside)	nt	nt	0.089	0.065
Eriocitrin (eriodictyol-7-*O*-rutinoside)	nd^b^	1.250	0.846	0.600
Hesperidin (hesperitin-7-*O*-rutinoside)	nt	1.870	2.049	0.935
Phloretin-3,5-di-C-glucoside	nt	1.270^*d*^	1.278	0.939
Aspalathin (3-hydroxyphloretin-3',5'-di-*C*-hexoside)	nt	nt	0.700	0.582
Iriflophenone-3-*C*-β-glucoside	nt	nt	0.669	0.590
Iriflophenone-di-*O*,*C*-hexoside	nt	nt	0.958	0.896
Protocatechuic acid	nt	nt	0.113	0.082

^a^Not tested

^b^Not detected

^c^Previously “Unknown 1” was quantified as luteolin equivalents as it appeared to be a flavone.

^d^Previously “Unknown 2” was quantified as hesperidin equivalents as it appeared to be a flavanone.

### Methanol extracts of *Cyclopia* act as agonists of ERβ, while all extracts antagonize E_2_-induced activation via ERα

**Figure 1:**
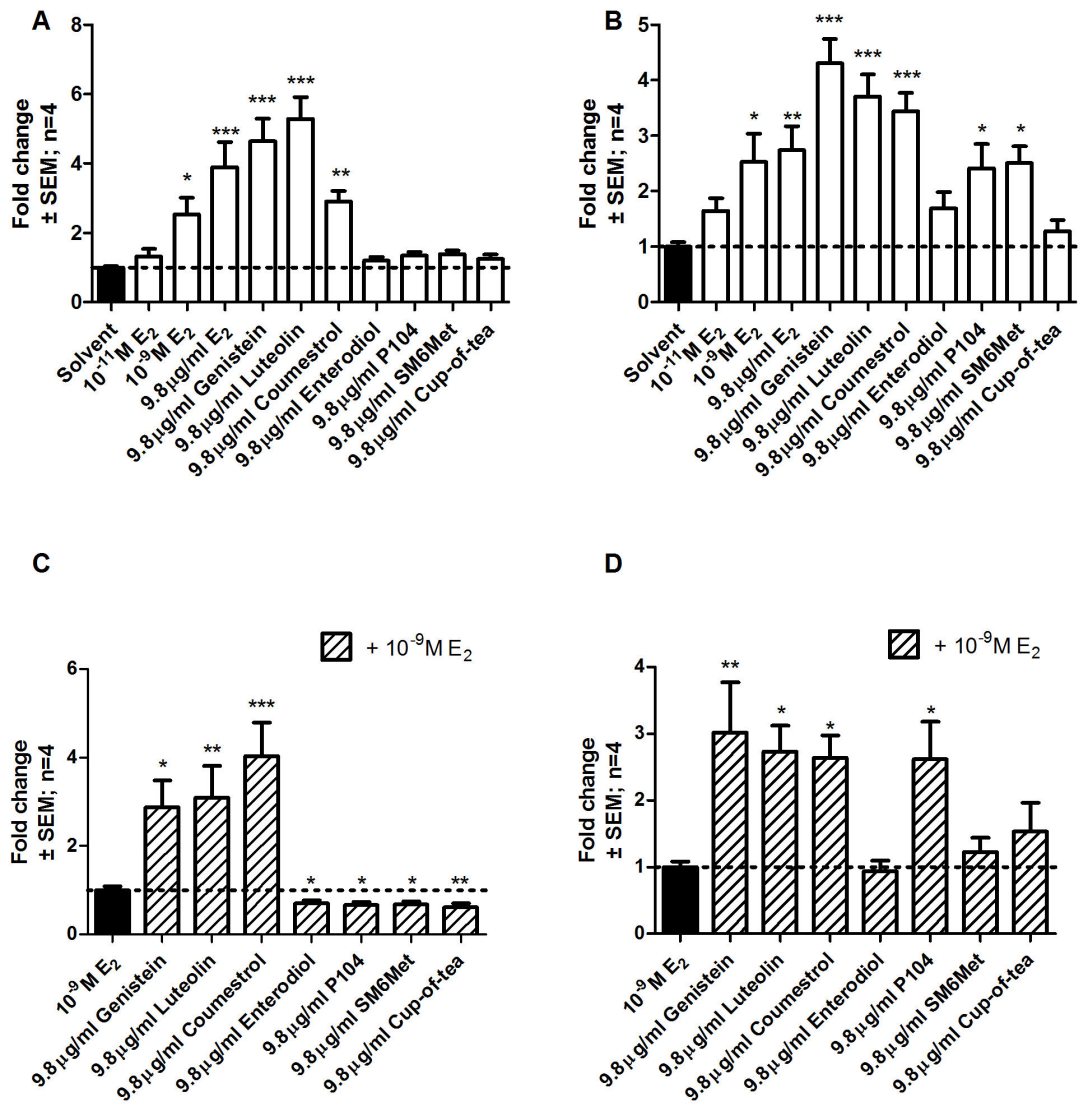
Evaluation of ER subtype specific agonism and antagonism of transactivation of an ERE-containing promoter reporter construct in COS-1 cells. COS-1 cells were transfected with either (A and C) pSG5-hERα or (B and D) pSG5-hERβ and ERE.vit2.luc and treated for 24 hours with a series of test compounds or extracts. To test agonism cells were treated with test compounds or extracts alone, (A and B), while, to test for antagonism cells were treated with test compounds or extracts in the presence of 10^-9^M E_2_ (C and D). Statistical analysis was done using One-way ANOVA with Dunnett’s post-test comparing all columns to the solvent control (*, P<0.05; **, P<0.01; ***, P<0.001). The dotted line through the bars represents the values for solvent control. Average ± SEM is of four independent experiments done in triplicate.

To evaluate ERα antagonism while also re-evaluating ERβ agonism COS-1 cells were transiently transfected with either ERα (Figures 1 A, C) or ERβ (Figures 1 B, D) and an ERE-containing promoter reporter construct. Agonism was tested in the absence (Figures 1 A, B) and antagonism in the presence (Figures 1 C, D) of 10^-9^ M E_2_. Three *Cyclopia* extracts, from two species, *C. genistoides* and *C. subternata*, were tested. Two were methanol extracts, P104 and SM6Met, and one was a water extract, cup-of-tea. In addition we investigated an example from each of the major classes of phytoestrogens: genistein, a well-studied isoflavone, enterodiol, a lignin, and coumestrol, a coumestan [84,85]. Luteolin, an estrogenic polyphenol [71], was also included as it was found be present in all of the *Cyclopia* extracts (Table 1), while E_2_ represents the major endogenous estrogen [86,87].

E_2_ induced ERα mediated transactivation in a dose dependent manner with significant induction at two concentrations of E_2_, 10^-9^ M (2.7 x 10^-4^ μg/ml) (2.5 ± 0.5 fold) and 9.8 µg/ml (3.6 x 10^-5^ M) (3.9 ± 0.7 fold), but not at the lowest concentration of 10^-11^ M (2.7 x 10^-6^ µg/ml) (Figure 1A). The same trend was seen for ERβ (2.5 ± 0.5 fold at 10^-9^ M and 2.7 ± 0.4 fold at 9.8μg/ml) (Figure 1B), although at the highest concentration of E_2_ higher induction was observed via ERα than via ERβ (3.9 ± 0.7 vs. 2.7 ± 0.4 fold). Although the 9.8 μg/ml E_2_ represents a supra-physiological concentration the 10^-11^ M and 10^-9^ M E_2_ concentrations reflect the pre- and post-menopausal levels of E_2_ respectively [88]. At the concentration of 9.8 µg/ml, genistein (3.6 x 10^-5^ M), luteolin (3.4 x 10^-5^ M), and coumestrol (3.7 x 10^-5^ M) significantly activated gene transcription through both of the ER subtypes (Figures 1A, B). Enterodiol, however, could not significantly activate gene transcription through either of the subtypes at the concentration of 9.8 µg/ml (3.2 x 10^-5^ M) (Figures 1A, B). None of the *Cyclopia* extracts were able to induce activation through ERα (Figure 1A), but both the methanol extracts, P104 and SM6Met, were able to significantly activate transcription through ERβ (2.4 ± 0.4 and 2.5 ± 0.3 fold, respectively).

To address antagonism, transactivation in the presence of 10^-9^ M E_2_ was evaluated (Figure 1C and D). The phenolic compounds, genistein, luteolin, and coumestrol were not antagonists but had an additive effect on E_2_-induced activation via both receptor subtypes (Figures 1C and D), confirming their agonism through both subtypes (Figure 1A and B). Enterodiol in contrast, however, only displays ERα antagonism (0.7 ± 0.1 fold vs. E_2_ activation set as 1) (Figure 1C). All of the *Cyclopia* extracts significantly antagonized ERα mediated E_2_-induction (P104, 0.7 ± 0.1, SM6Met, 0.7 ± 0.1, and cup-of-tea, 0.6 ± 0.1 fold), however, only P104 had an additive effect on the E_2_-induced activation through ERβ (Figure 1D). In conclusion, the methanol extracts of *Cyclopia* are ERβ agonists and all extracts are ERα antagonists.

### In MCF-7BUS cells expressing both ER subtypes all extracts of *Cyclopia transactivate* an ERE-driven promoter reporter construct

Most tissues affected by menopause and/or implicated in HRT side effects, such as uterus, bone, and breast, contain both ER subtypes [89]. Thus, having shown that methanol extracts of *Cyclopia* are ERβ agonists and all extracts are ERα antagonists in a system where the ER subtypes were evaluated separately, we were interested in investigating the transactivation potential of *Cyclopia* extracts in a system where both subtypes are present. 

**Figure 2:**
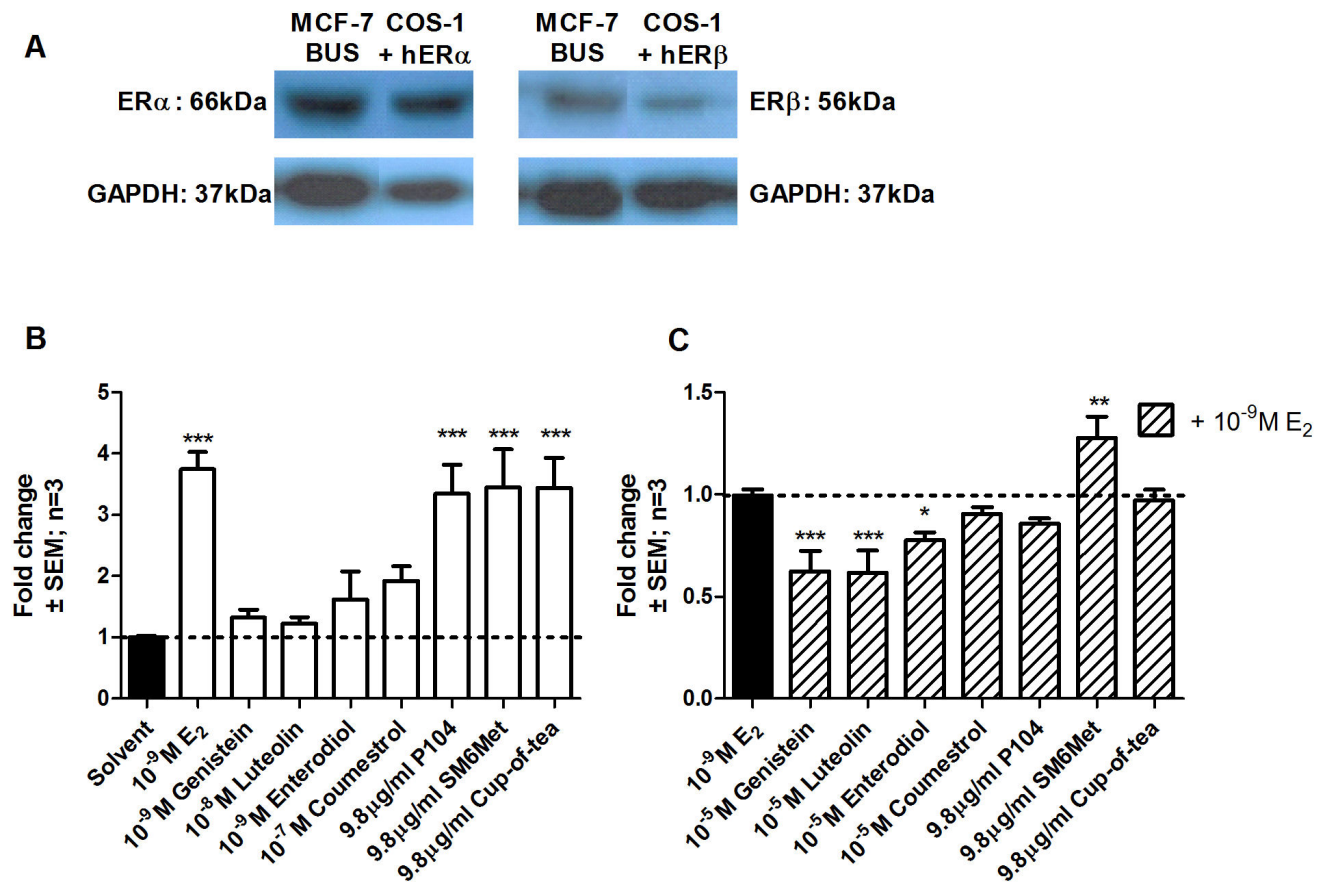
Evaluation of transactivation of an ERE-containing promoter reporter construct in MCF-7BUS cells expressing both ERα and ERβ. MCF-7BUS cells, with endogenous ERα and ERβ (A), were transfected with ERE.vit2.luc and treated for 24 hours with a series of test compounds or extracts. To test agonism cells were treated with test compounds or extracts alone (B), while, to test for antagonism cells were treated with test compounds or extracts in the presence of 10^-9^M E_2_ (C). Statistical analysis was done using One-way ANOVA with Dunnett’s post-test comparing all columns to the solvent control (*, P<0.05; **, P<0.01; ***, P<0.001). The dotted line through the bars represents the values for solvent control. Average ± SEM is of three independent experiments done in triplicate.

MCF-7BUS cells, containing both ERα and ERβ (Figure 2A), were transfected with an ERE-containing promoter reporter construct and both agonism (Figure 2B) and antagonism (Figure 2C) were tested. Although strong transactivation was seen with E_2_, none of the polyphenols on their own were able to significantly activate gene transcription in this system where both ER subtypes are present (Figure 2B), despite the fact that these polyphenols transactivate when the ER subtypes function in isolation (Figure 1A and B). Furthermore, most of the polyphenols, excluding coumestrol, antagonized E_2_ induction when both ER subtypes are together (Figure 2C), whereas when the subtypes were expressed separately only enterodiol showed ERα antagonism (Figure 1C). In contrast to the polyphenols, the extracts of *Cyclopia*, P104 (3.4 ± 0.5 fold), cup-of-tea (3.4 ± 0.5 fold) and, SM6Met (3.5 ± 0.6 fold), were able to activate transcription to a similar extent as 10^-9^ M E_2_ (3.8 ± 0.3 fold) (Figure 2B). These results, together with the fact that the *Cyclopia* extracts did not antagonize E_2_ induction (Figure 2C), suggests that when both ER subtypes are co-expressed the *Cyclopia* extracts act as agonists, whereas when the ER subtypes are expressed separately they only act as agonists through ERβ and antagonize ERα induction. 

An extract of *C. genistoides* represses NFκB activation via ERα and ERβ whereas the extracts of *C. subternata* are ERβ antagonists.

**Figure 3:**
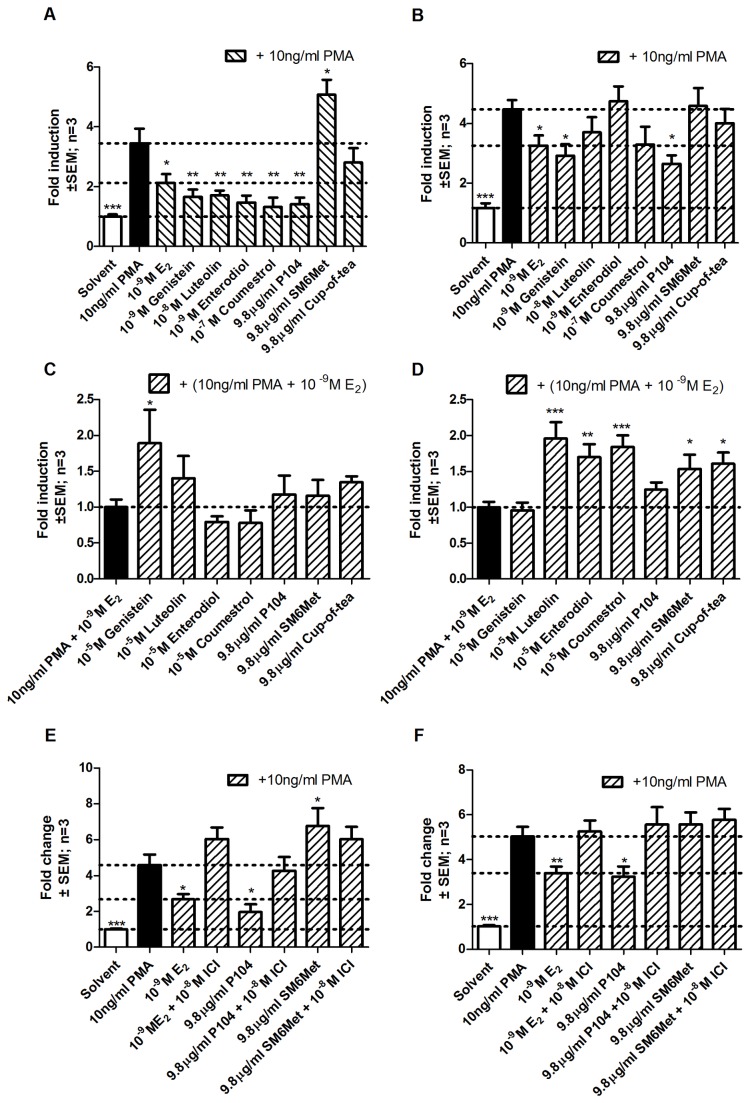
Evaluation of ER subtype specific agonism and antagonism of an NFκB-containing promoter reporter construct in COS-1 cells. COS-1 cells were transfected with either (A, C, and E) pSG5-hERα or (B, D, and F) pSG5-hERβ and p(IL6kB)350hu.IL6Pluc+ and treated for 24 hours with a series of test compounds or extracts. To test agonism cells were treated with test compounds or extracts alone, (A and B), while, to test antagonism cells were treated with test compounds or extracts in the presence of 10^-9^ M E_2_ (C and D). To ascribe the observed effect to the ER we treated cells with P104 and SM6Met in the absence or presence of the ER antagonist ICI 182,870 (E and F). Statistical analysis was done using One-way ANOVA with Dunnett’s post-test comparing all columns to either (A, B, E, and F) 10ng/ml PMA or (C and D) 10ng/ml PMA + 10^-9^M E_2_ (*, P<0.05; **, P<0.01; ***, P<0.001). The dotted lines through the bars represent the values for either (A, B, E, and F) solvent control, 10ng/ml PMA, or 10ng/ml PMA + 10^-9^M E_2_ or (C and D) 10ng/ml PMA + 10^-9^M E_2_. Average ± SEM is of three independent experiments done in triplicate.

The decline in estrogen levels during menopause leads to a surge in the occurrence of inflammatory disorders [52,90-92]. Furthermore, NFκB, a pro-inflammatory transcription factor, is involved in the development of breast cancer [93-95]. Taking this into account we wanted to evaluate the ability of *Cyclopia* extracts to repress the activation of an NFκB-containing promoter reporter construct by transfecting COS-1 cells with said construct and either ERα (Figures 3A, C, E) or ERβ (Figures 3B, D, F). In addition, this system would provide information concerning the behavior of *Cyclopia* extracts in a transrepression model. Agonism was tested in the absence (Figures 3A, B) and antagonism (Figures 3C, D) in the presence of 10^-9^ M E_2_. 

PMA (phorbol 12-myristate 13-acetate, an activator of NFκB driven gene expression [96,97]) activation of the NFκB-containing construct was repressed by E_2_ via both receptor subtypes (Figure 3A and B) with a more pronounced repression through ERα (38.6% vs. 27.2%). Like E_2_, all of the polyphenols, as well as P104 (*C. genistoides* extract), acted as ERα agonists by repressing PMA activation (genistein 52.1%, luteolin 50.6%, enterodiol 57.4%, coumestrol 61.8%, and P104 59.2%) (Figure 3A). Furthermore, genistein (34.8% repression) and P104 (40.7% repression), like E_2_, also displayed significant ERβ agonism (Figure 3B). Therefore, in our transrepression model P104 is not an ERβ selective agonist, but displays agonism via both subtypes. The water extract of *C. subternata*, cup-of-tea, was unable to repress PMA induction through either ERα or ERβ (Figures 3A, B) while the methanol extract, SM6Met, also unable to repress PMA induction through either subtype, significantly added to the activation observed with PMA alone via ERα (5.1 ± 0.5 vs. 3.5 ± 0.5) (Figure 3A, B).

Antagonism was evaluated in the presence of 10^-9^ M E_2_ and only genistein (Figure 3C) had a significant effect via ERα by antagonizing E_2_ repression of PMA activation. The polyphenols, luteolin, enterodiol, and coumestrol, but not genistein, however, antagonized E_2_ repression of PMA activation via ERβ (Figure 3D). Although none of the extracts displayed significant antagonism of ERα, the extracts of *C. subternata* displayed ERβ antagonism (Figure 3D).

The result for SM6Met in Figure 3A prompted us to investigate whether this effect was via ERα or if SM6Met is able to activate the NFκB-containing construct through another mechanism of action. Therefore, we repeated the experiment, for both receptor subtypes, with SM6Met, as well as P104, in the presence and absence of an ER antagonist, ICI 182,780 (Figures 3E, F). The observed repression of PMA activation by E_2_ and P104 via ERα and ERβ is abolished by ICI (Figure 3E, F) and thus, the observed repression is indeed via the ER. SM6Met, like ICI, increases PMA activation through ERα (Figure 3E) and both have no significant effect on PMA activation via ERβ (Figure 3F). Furthermore, the increased transactivation observed with SM6Met in Figure 3A may be attributed to residual E_2_ remaining after stripping of FCS, as suggested by others [22], which would further support the contention that SM6Met is behaving as an ERα antagonist. In conclusion then the results suggest that for our transrepression model the methanol extract of *C. genistoides* (P104) is behaving like an ERα and ERβ agonist, while the methanol extract of *C. subternata* (SM6Met) is an ERα antagonist in the absence of E_2_, and an ERβ antagonist in the presence of E_2_.

In MCF-7BUS cells expressing both ER subtypes all extracts are agonists, while the water extract of *C. subternata* also displays antagonistic activity.

As we have shown that P104 is an ER agonist and SM6Met is an ER antagonist in a transrepression model where the ER subtypes function in isolation (Figure 3), we wanted to test the effect of these extracts in a model where both subtypes are present as most tissues affected by menopause and/or implicated in HRT side effects contain both subtypes.

**Figure 4:**
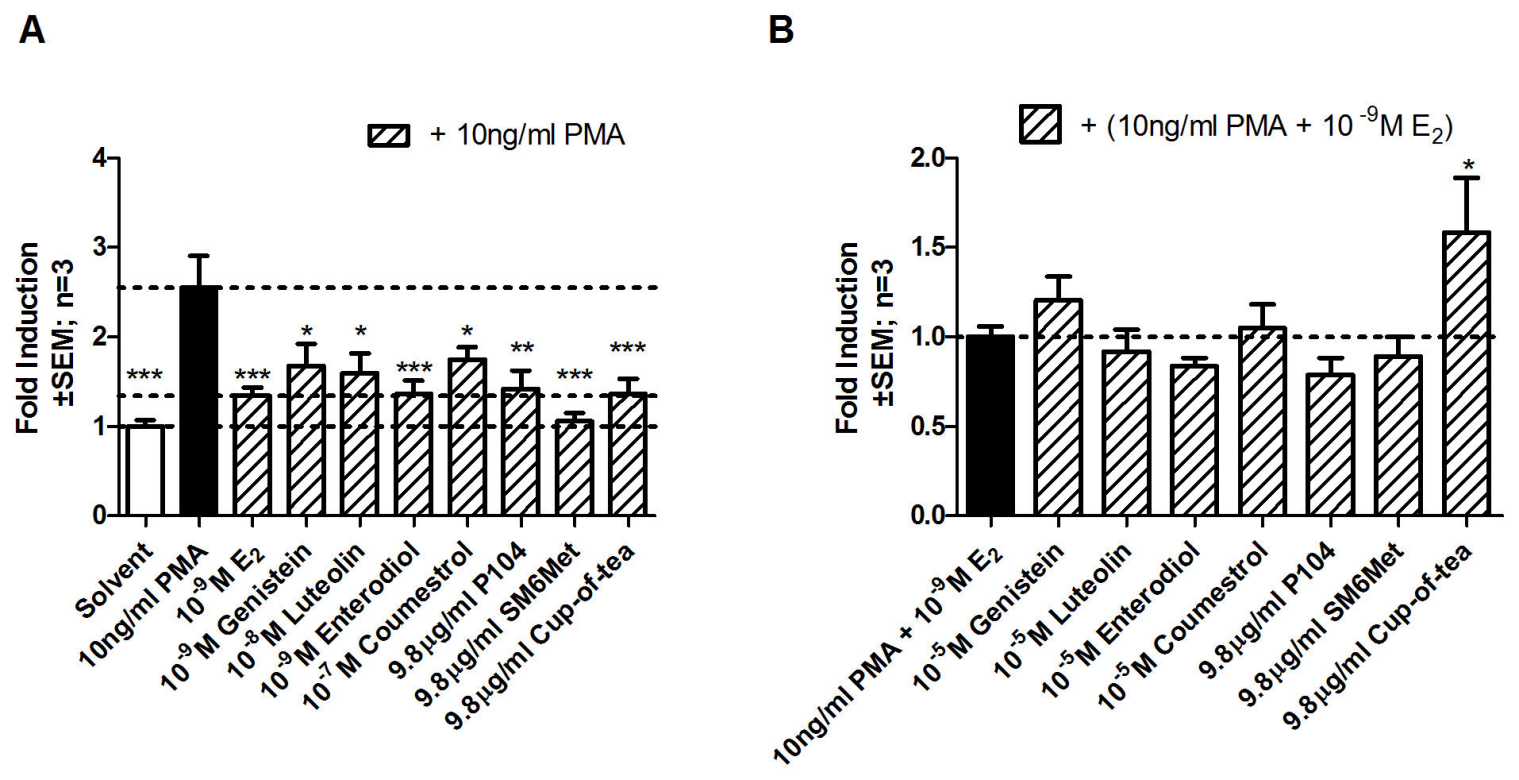
Evaluation of transrepression of an NFκB-containing promoter reporter construct in MCF-7BUS cells expressing both ERα and ERβ. MCF-7BUS cells were transfected with p(IL6kB)350hu.IL6Pluc+ and treated for 24 hours with a series of test compounds or extracts. To test agonism cells were treated with test compounds or extracts alone, (A), while, to test for antagonism cells were treated with test compounds or extracts in the presence of 10^-9^M E_2_ (B). Statistical analysis was done using One-way ANOVA with Dunnett’s post-test comparing all columns to either (A) 10ng/ml PMA or (B) 10ng/ml PMA + 10^-9^M E_2_ (*, P<0.05; **, P<0.01; ***, P<0.001). The dotted lines through the bars represent the values for either (A) solvent control, 10ng/ml PMA, or 10ng/ml PMA + 10^-9^M E_2_ or (B) 10ng/ml PMA + 10^-9^M E_2_. Average ± SEM is of three independent experiments done in triplicate.

MCF-7BUS cells were transfected with an NFκB-containing promoter reporter construct and both agonism (Figure 4A) and antagonism (Figure 4 B) evaluated. Strong repression was observed with E_2_, the polyphenols, and P104 when both subtypes are present (Figure 4A), which correlates with what was observed previously for ERα alone (Figure 3A). However, for ERβ alone (Figure 3B), significant repression was previously seen only with E_2_, genistein, and P104 but not with luteolin, enterodiol, and coumestrol. Unlike previous results, SM6Met behaved differently when subtypes were co-expressed than when the subtypes were expressed separately. It displayed agonism when subtypes are expressed together (Figure 4A) while displaying antagonism when expressed separately (Figure 3A and D). Similarly, where no agonist activity via either subtype alone was observed previously, the cup-of-tea extract was able to change its behavior when both subtypes are present by displaying ER agonism. Furthermore, antagonism in the presence of both subtypes was only seen with the cup-of-tea extract (Figure 4B), while the subtype specific antagonism of genistein, luteolin, enterodiol, coumestrol, and SM6Met (Figures 3C, D) is abrogated in the presence of both subtypes. Taken together, in a transrepression model, the DME of *C. genistoides*, P104, is an ER agonist in all models (Figures 3A, B, and 4A), the DME of *C. subternata*, SM6Met, is an ERβ antagonist in the presence of E_2_ (Figure 3D), an ERα antagonist in the absence of E_2_ (Figure 3A, E), and an agonist in the presence of both ER subtypes (Figure 4A), while the water extract of *C. subternata*, cup-of-tea, is an ERβ antagonist (Figure 3D) and an ER agonist/antagonist (Figures 4A, B) in the presence of both subtypes. This differential behavior of the *Cyclopia* extracts in the transrepression model contrasts to similar behavior by the extracts in the transactivation model where all extracts displayed antagonism through ERα (Figure 1) alone, while displaying agonism to ERβ (Figure 1) alone or when both subtypes are expressed (Figure 2).

### *Cyclopia extracts* weakly induce proliferation of breast cancer cells but antagonizes E_2_-induced breast cancer cell proliferation

**Figure 5:**
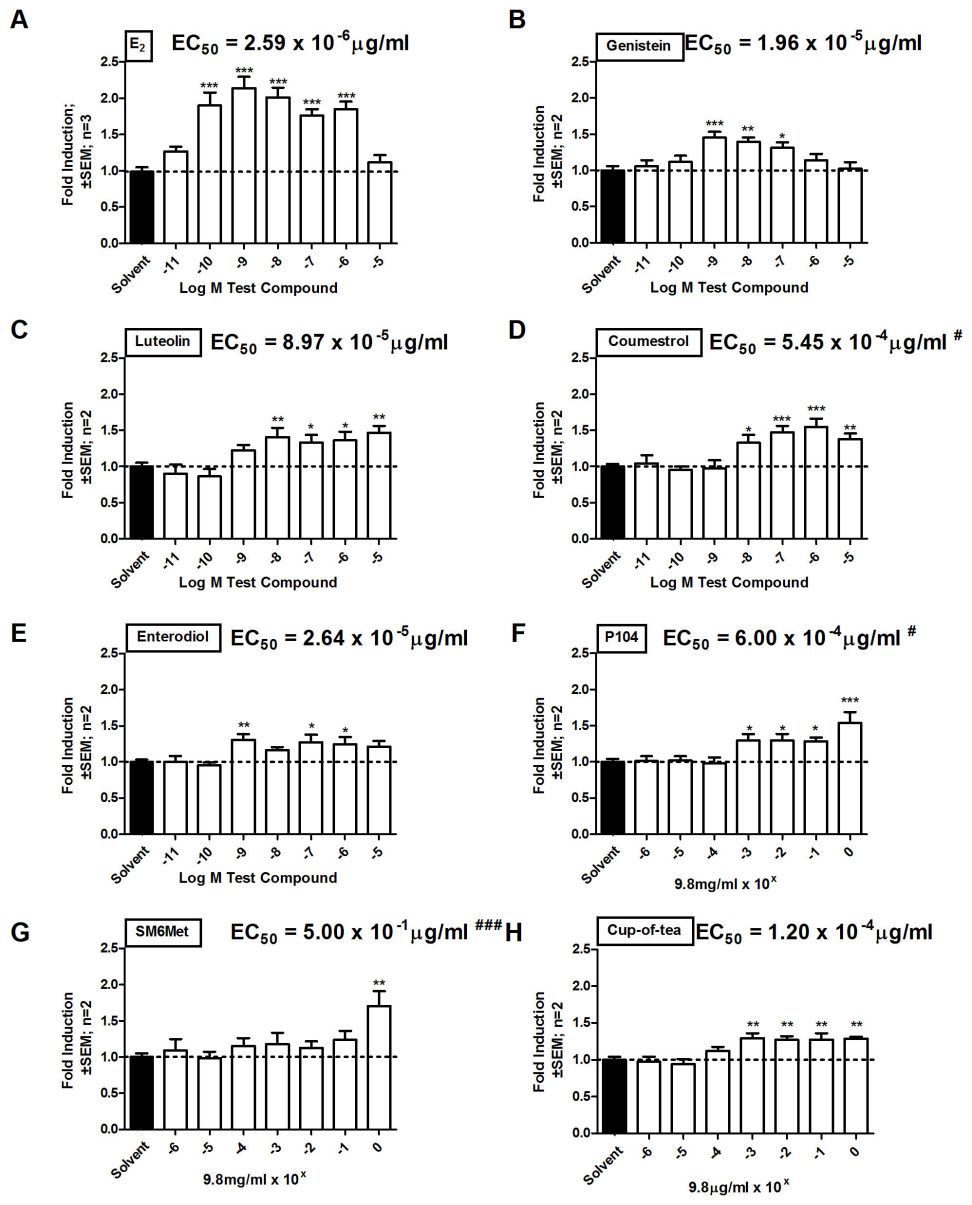
Evaluation of agonism of proliferation, a more complex endpoint encompassing both transactivation and transrepression in MCF-7BUS cells expressing both ERα and ERβ. MCF-7 BUS cells were treated with increasing concentrations of (A) E_2_, (B-E) polyphenols, and (F-H) *Cyclopia* extracts for 48 hours. After treatment the amount of living cells was determined using a MTT assay. Statistical analysis was done using One-way ANOVA with Dunnett’s post-test comparing all columns to the solvent control (*, P<0.05; **, P<0.01; ***, P<0.001) or to E_2_ for EC_50_ values (#, P<0.05; ##, P<0.01; ###, P<0.001). The dotted line through the bars represents the values for solvent control. Average ± SEM is of two independent experiments done in six replicates, except (A) where average ±SEM is of three independent experiments done in six replicates.

**Figure 6:**
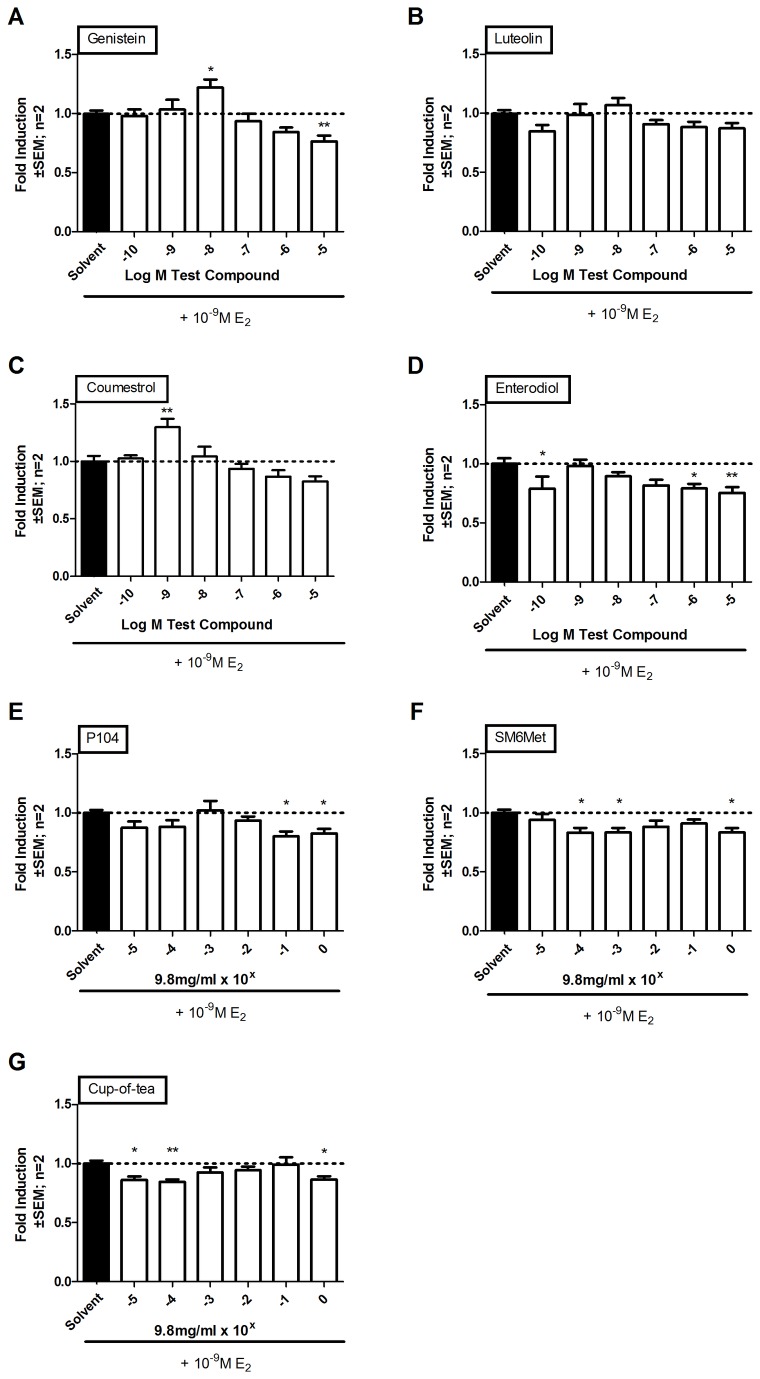
Evaluation of antagonism of proliferation, a more complex endpoint encompassing both transactivation and transrepression in MCF-7BUS cells expressing both ERα and ERβ. MCF-7 BUS cells were treated with increasing concentrations of (A-D) polyphenols and (E-G) *Cyclopia* extracts for 48 hours in the presence of 10^-9^M E_2_. After treatment the amount of living cells was determined using a MTT assay. Statistical analysis was done using One-way ANOVA with Dunnett’s post-test comparing all columns to the solvent control (*, P<0.05; **, P<0.01; ***, P<0.001). The dotted line through the bars represents the values for solvent control. Average ± SEM is of two independent experiments done in six replicates.

Having shown that *Cyclopia* extracts can modulate both transactivation and transrepression in the presence of both ER subtypes and when the subtypes are expressed alone, we wanted to re-evaluate agonism of P104 [63], SM6Met and cup-of-tea [68] (Figure 5) and antagonism of P104 [63] (Figure 6) and for the first time evaluate antagonism of SM6Met and cup-of-tea (Figure 6) on MCF-7BUS breast cancer cell proliferation. Cell proliferation in MCF-7BUS cells constitutes an integrated model where not only the ER subtypes are co-expressed, but both transactivation and transrepression of endogenous genes contribute towards the final phenotype, whether it is proliferative or anti-proliferative [39,98-100]. 

The MTT cell proliferation assay using MCF-7BUS cells was used to address agonism (Figure 5A-H). Estrogen induced cell proliferation at a wide range of concentrations (10^-6^ M to 10^-10^ M) with the highest efficacy (2.1 ± 0.1 fold) observed at 10^-9^ M E_2_ (2.7 x 10^-4^ μg/ml) (Figure 5A). Like E_2_, all of the polyphenols were also able to induce cell proliferation, but not to the same extent as E_2,_ with a maximum efficacy of: genistein, 1.5 ± 0.1 fold at 10^-9^ M (2.7 x 10^-4^ μg/ml) (Figure 5B), luteolin, 1.5 ± 0.1 fold at 10^-5^ M (2.7 μg/ml) (Figure 5C), coumestrol, 1.6 ± 0.1 fold at 10^-6^ M (3.0 x 10^-1^ μg/ml) (Figure 5D), and enterodiol, 1.3 ± 0.1 fold at 10^-9^ M (3.0 x 10^-4^ μg/ml) (Figure 5E). Similarly, all three extracts of *Cyclopia* induced proliferation of cells with a lower efficacy than E_2_ with maximum efficacies of 1.5 ± 0.2 (significantly different from E_2_), 1.3 ± 0.03 (significantly different from E_2_), and 1.7 ± 0.2 (not significantly different from E_2_) fold for 9.8 μg/ml of P104, cup-of-tea and SM6Met, respectively (Figures 5F-H). The potencies, depicted by EC_50_ values on graphs (Figures 5A-H), of the polyphenols, as well as of the *Cyclopia* extracts, were lower than that of E_2_ with coumestrol, P104, and SM6Met significantly lower and may be listed in order of decreasing potency as follow: E_2_ > genistein > enterodiol > luteolin > cup-of-tea > P104 > coumestrol >> SM6Met. 

To address antagonism (Figure 6A-G), increasing concentrations of the polyphenols and *Cyclopia* extracts were tested in the presence of 10^-9^ M E_2_ (highest efficacy, Figure 5A). Genistein (Figure 6A) and enterodiol (Figure 6D), significantly repressed E_2_-induced cell proliferation (23.3% at 10^-5^ M (2.70 μg/ml) and 24.5% at 10^-5^ M (3.02 μg/ml), respectively). Although, luteolin (Figure 6B) and coumestrol (Figure 6C) displayed no significant antagonism, coumestrol did have a significant additive effect (1.3 ± 0.1 fold) at 10^-9^ M (2.96 x 10^-4^ μg/ml), suggesting agonism. Similarly, genistein, an antagonist at high concentrations, also had a significant additive effect (1.2 ± 0.1 fold) at the lower concentration of 10^-8^ M (2.70 x 10^-3^ μg/ml) (Figure 6A). All extracts of *Cyclopia* were able to antagonize E_2_-induced cell proliferation, with P104 repressing 19.8% at 9.8 x 10^-1^ μg/ml, SM6Met 16.8% 9.8 x 10^-4^ μg/ml, and cup-of-tea 15.6% repression at 9.8 x 10^-4^ μg/ml (Figures 6E, F, G). Taken together, these results show that although all extracts of *Cyclopia* induced cell proliferation, the P104 and cup-of-tea extracts did so at a significantly lower efficacy and the methanol extracts at a significantly lower potency than E_2,_ and that all extracts could antagonize E_2_-induced cell proliferation.

### SM6Met does not stimulate the growth of rat uteri, antagonizes E_2_-induced uterine proliferation, and delays vaginal opening

**Figure 7:**
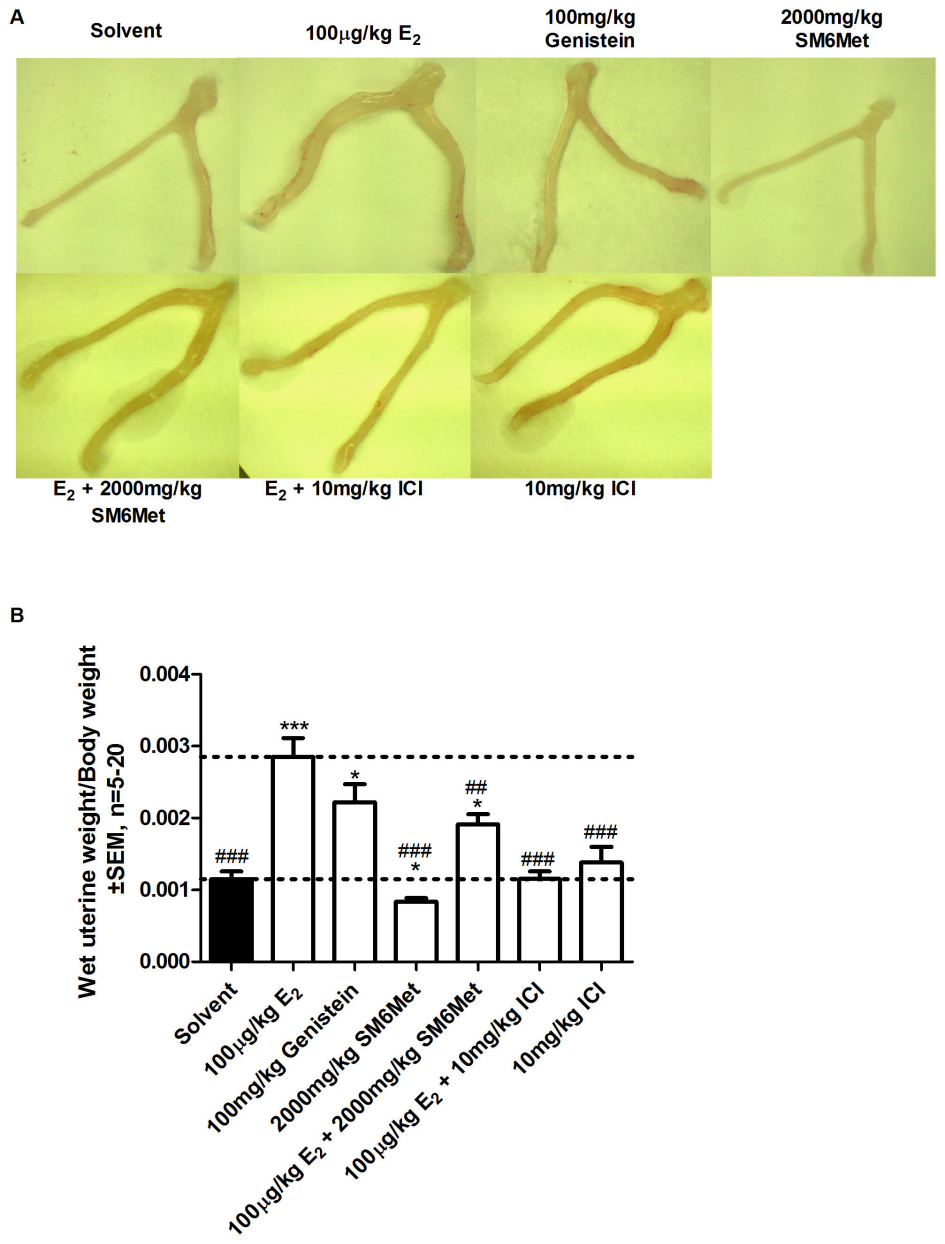
Evaluation of the *in* *vivo* effect of E_2_, genistein and SM6Met on immature rat uterine growth. Immature female wistar rats were treated with 100µg/kg body weight E_2_, in the presence and absence of 2000mg/kg body weight SM6Met or 10mg/kg body weight ICI 182,780, 100mg/kg body weight genistein, 2000mg/kg body weight SM6Met, and 10mg/kg body weight ICI 182,780 for three consecutive days. Animals were sacrificed on day four, (A) uteri were photographed, and (B) wet uterine/final body weight was determined. One-way ANOVA with Dunnett’s post-test comparing all columns to either solvent control (*, P<0.05; **, P<0.01; ***, P<0.001) or E_2_ (#, P<0.05; ##, P<0.01; ###, P<0.001). The dotted lines through the bars represent the values for solvent control or E_2_. Average ± SEM is of at least five animals/group.

For the *in vivo* studies only extracts from *C. subternata* was used as P104 plant material was not available in bulk. The immature rat uterotrophic assay is used to determine the ability of test compounds to stimulate ERα induced uterine growth as ERβ is not highly expressed in the uterus [56,101] and also allows for the detection of antiestrogens [102]. Rats were administered E_2_, genistein, and the two *C. subternata* extracts, SM6Met and cup-of-tea, via oral gavage and the effects on uterine growth were evaluated (Figure 7A, B, and Figure S3). Estrogen, as well as genistein, induced uterine growth (2.5 ± 0.2 and 2.0 ± 0.2 fold, respectively) (Figure 7). In contrast, the extracts significantly reduced uterine weight relative to solvent (Figure 7 and Figure S3). SM6Met also significantly repressed E_2_-induced uterine growth by 33.0%, a result that is similar, but less pronounced, than that seen with ICI 182,780 (59.7% repression) (Figure 7) suggesting that the extracts behave as antiestrogens in the uterus. 

We also addressed body weight changes and toxicity (Figure S4) and found that E_2_ significantly increased body weight, whereas genistein significantly decreased body weight. The extracts of *Cyclopia* and ICI 182,780, however, did not lead to significant weight gain or loss as compared to solvent, except for the animals treated with the highest concentrations (2000mg/kg BW) of SM6Met and cup-of-tea extracts which gained significantly less weight than the solvent treated animals. With regards to toxicity, none of the treated animals showed any significant changes in liver weight, except for a decrease in liver weight in animals treated with 200mg/kg BW SM6Met.

**Figure 8:**
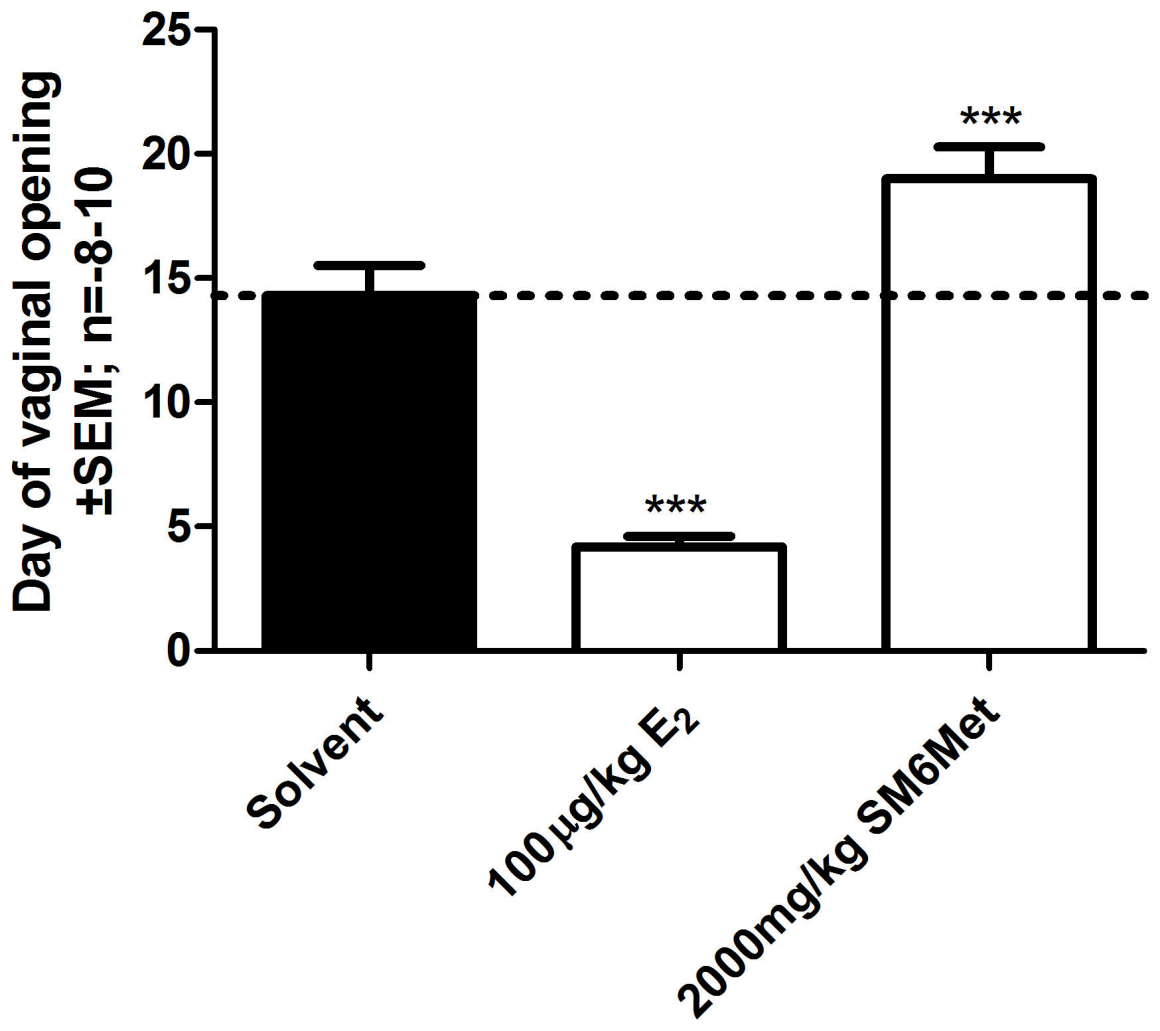
Evaluation of the effect of E_2_ and SM6Met on the timing of vaginal opening. Immature female wistar rats were treated for 30 consecutive days with 100μg/kg body weight E_2_ and 2000mg/kg body weight SM6Met and the day of vaginal opening was determined. One-way ANOVA with Dunnett’s post-test comparing all columns to solvent control (*, P<0.05; **, P<0.01; ***, P<0.001). The dotted line through the bars represents the values for solvent control. Average ± SEM is of at least eight animals/group.

Furthermore, as another marker of estrogenic activity, albeit a less sensitive marker [102], we also evaluated time of vaginal opening over an extended period of daily treatments (Figure 8). Estrogen led to premature vaginal opening when compared to solvent (4.2 ± 0.4 vs. 14.3 ± 1.2 days). This correlates with the observed increase in uterine weight in Figure 7. The significantly delayed vaginal opening of SM6Met treated animals (19.0 ± 1.3 days) also correlates with uterine weight results in displaying antiestrogenic behavior. The significant delay in vaginal opening was observed for all three of the concentrations of SM6Met, however, although the cup-of-tea extract showed a similar trend, it was not significant (Figure S5).

To summarize, for the first time we show that the *C. subternata* extracts are absorbed when administered orally and elicit a biological effect *in vivo*. Specifically, *Cyclopia* extracts, in contrast to E_2_ and genistein, did not induce uterine growth and SM6Met antagonized E_2_-induced uterine growth. Furthermore, the extracts also delayed vaginal opening in contrast to E_2_. These results suggest that the *Cyclopia* extracts display ERα antagonism *in vivo* by retarding uterine growth [56,101].

## Discussion

HRT in the form of estrogens provides relief from the plethora of menopause associated symptoms [1]. Although these estrogens provide relief from menopausal symptoms, they introduced new HRT associated risks, including an increased occurrence of breast cancer, heart disease, strokes, and endometrial cancer [1,5,6,8]. These risks, and the associated reluctance of usage, instigated the search for a new generation of estrogen analogues that would provide the benefits of estrogens without the associated risks. In addition, it would be of great value if these new analogues display chemo-preventative properties in breast and endometrial tissues [9,10,29].

The search for new estrogen analogues heralded the era of the SERMs. These SERMs would selectively modulate estrogen receptors in different tissues, acting as antagonists in the breast and uterus (chemo-preventative) and as agonists in the bone (osteoporosis prevention). Tamoxifen, a first generation SERM, provided the desired protective effect in the breast [31,32] and raloxifene, a second generation SERM, had protective properties in breast and bone tissues [26,27,103]. However, as these SERMS have been linked to the increased occurrence of hot flashes and stimulated endometrial growth (tamoxifen), the search continues [28,34,35]. Third generation SERMs, such as lasoxifene and bazedoxifene, are currently in development, but the focus has shifted to osteoporosis treatment with protection against breast cancer as a beneficial side effect [104-106].

Although SERM development continues there is increased interest in SERSMs, analogues that can differentially modulate specific ER subtypes. This was brought on by studies that have shown that ERβ inhibits ERα dependent cell proliferation and could prevent cancer development [15,22,37,40-43]. Phytoestrogens have been shown to be both estrogenic as well as antiestrogenic and while they can bind to both ER subtypes, they generally have a higher affinity for ERβ as well as a higher transcriptional potency and efficacy via ERβ [61-63]. Thus, phytoestrogen rich food sources have become important potential resources of SERSMS.

The current study evaluated previously described extracts of *Cyclopia*, a source of phytoestrogens, for ER agonism and/or antagonism (summarized in Table S1). Specifically, we evaluated the effect of *Cyclopia* extracts on transactivation and transrepression in a model where ERα and ERβ were expressed separately. This allows for the evaluation of the modulation of ER subtype specific activity in two transcriptional models: a classical ERE transactivation model and an NFκB transrepression model. In the transactivation model the methanol extracts, P104 and SM6Met were ERβ agonists, while all extracts antagonized ERα. In the transrepression model, however, the behavior of the *Cyclopia* extracts became more complex. P104, which displayed opposite effects via the subtypes in the transactivation model, acted as an agonist for both subtypes in the transrepression model. The extracts of *C. subternata*, however, did not elicit such uniform effects in the transrepression model. SM6Met, a methanol extract, acting as an ERα antagonist and ERβ agonist regarding transactivation, displayed antagonism towards ERα, in the absence of E_2,_ and towards ERβ, in the presence of E_2_. Similar antagonism towards ERα in the absence of E_2_ has also been seen for the plant extract MF101 regarding IL6 mRNA expression [24]. The water extract, cup-of-tea, also changed its behavior, acting as an ERβ antagonist for transrepression as opposed to an ERα antagonist for transactivation. These behavioral changes were not exclusive to the *Cyclopia* extracts as the polyphenols also displayed these characteristics. Luteolin, for example, displayed ER agonism through both subtypes in the transactivation model but was an ERα agonist and an ERβ antagonist in the transrepression model. The occurrence of mixed agonism and antagonism towards ER subtypes has also been observed for the xenoestrogen, Bisphenol A (BPA) [107]. 

As the current experiments were performed in the same cell line we have to look towards differences between the mechanisms of transactivation and transrepression for clarification of these results. Classically, transactivation is a product of ER dimer binding directly to the DNA sequence, however, tethering to DNA bound transcription factors (TFs) in the promoter region of affected genes has also been described [108-111]. Binding of the ER to DNA, whether it is direct or indirect, initiates the recruitment of co-activators, which then modulates transcription [112]. Regarding transrepression, specifically the repression of NFκB driven genes, various mechanisms of ER mediated transrepression have been described [109]. The ER can bind to NFκB and thereby prevent DNA binding of the transcription factor [113,114], ligand bound ER present at promoter regions can recruit co-repressors [115,116], ligand bound ERα and activated NFκB can compete for co-activator recruitment [117,118], or ERα, through a non-genomic pathway, inhibits translocation of activated NFκB to the nucleus [119]. We can use this knowledge of the mechanism of action and combine it with what we know about SERMs and ER subtypes specific ligands to postulate a mechanism of action of *Cyclopia* agonism and antagonism. For the SERMs, three mechanisms of antagonism have been proposed [18]. SERMs can bind to the ER with a higher affinity than E_2_ and block the binding of E_2_, they can block the binding of co-activators, or SERMs can induce the recruitment of co-repressors [18,120,121].. Not much is known regarding the mechanism of SERM agonism [18], although it has been suggested that they can block the binding of co-repressors [121]. In addition, MF101 and liquiritigenin, both ERβ selective agonists, although being able to bind to ERα, cannot recruit co-activators to ERα, and MF101 cannot promote the interaction of ERα with regulatory elements [15,24]. Furthermore, it has been suggested that SERMs may activate cell surface signaling pathways that results in ligand-independent activation of ERs [29,122,123].

Therefore, with regards to transactivation, we may postulate that the extracts of *Cyclopia* cannot transactivate via ERα as they are unable to recruit the necessary co-activators, while for ERβ, P104 and SM6Met are able to do so. It is also possible that the extracts of *Cyclopia* cannot induce ERα interaction with regulatory elements. The observed ERα antagonism of E_2_-induced transactivation may be due to the extracts binding to ERα and either inhibiting E_2_ binding, inhibiting the recruitment of co-activators or stimulating the recruitment of co-repressors. In our transrepression model P104 behaves like E_2_ and could be exerting its function by any of the NFκB repression models discussed earlier. However, SM6Met displays ERα antagonism in the absence of E_2_ and this antagonism is lost in the presence of E_2_. Therefore, it is possible that SM6Met is unable to recruit co-repressors in the absence of E_2_ and is unable to inhibit the E_2_-induced recruitment of co-repressors. Furthermore, antagonism of ERβ in the transrepression model by SM6Met and cup-of-tea may be due to the recruitment of co-activators to ERβ.

Next we evaluated agonism and antagonism of *Cyclopia* extracts in a more complex environment where the ER subtypes are co-expressed. We used the MCF-7BUS cells, a breast carcinoma cell line, not only because it co-expresses the subtypes (Figure 2A), but also to evaluate the activity of the extracts in breast tissue cells. With regards to transactivation, all extracts of *Cyclopia* were agonists and are likely exerting this agonism through ERβ as they were ERβ agonists and ERα antagonists in COS-1 cells. Also, previously we discussed the possibility that the extracts may be unable to recruit co-activators to ERα or induce ERα-regulatory element interactions, which supports the idea that the *Cyclopia* extracts are mediating their transactivative effects in MCF-7BUS cells via ERβ. Interestingly, the polyphenols, genistein and luteolin, having displayed ER agonism in COS-1 cells, in an environment where both ER subtypes are present displayed only weak agonism, which may be attributed to the fact that lower concentrations were used in MCF-7BUS cells. However, when both subtypes are present these polyphenols display antagonism, which was not apparent when the subtypes were expressed separately. When both ER subtypes are expressed in the transrepression model, all the polyphenols as well as the *Cyclopia* extracts acted as agonists, while the water extract of *C. subternata* also displayed ER antagonism. The ER agonism of P104 in the transrepression model is thus not a cell type selective effect as it is seen in both the COS-1 (kidney) and MCF-7BUS (breast) cells. The ER antagonism of cup-of-tea in MCF-7BUS cells is likely mediated via ERβ as ERβ antagonism was observed in COS-1 cells transfected with ERβ, but not in cells transfected with ERα. However, the SM6Met extract, which displayed antagonism for ERα and ERβ in COS-1 cells, changes its behavior in the MCF-7BUS cells and acts as an ER agonist in the transrepression model. Furthermore, a similar switch in behavior is observed with the polyphenols as the subtype specific antagonism is abrogated in the presence of both ER subtypes. These observed behavioral changes of the *Cyclopia* extracts as well as the polyphenols in different tissues have also been observed for the SERM, tamoxifen [18]. Ball et al. [18] found that tamoxifen differentially regulated ER regulated genes in different cell lines and ascribed this phenomenon to the presence, or lack of, co-regulators in different tissues. Therefore, the differential effect of *Cyclopia* extracts as well as the polyphenols in cells from different tissues might be due to changes in the co-regulator environment.

As MCF-7BUS cells express both ER subtypes, we also have to consider the possibility of ERα/β heterodimer formation and the biological relevance thereof as opposed to homodimer formation in COS-1 cells expressing the ER subtypes in isolation. Using two phytoestrogens that are ERα/β heterodimer selective, cosmosiin and angolensin, it was shown that heterodimer formation, in the presence of these ligands, leads to higher activation of an ERE-promoter reporter construct than homodimers and furthermore that heterodimer formation has a growth inhibitory effect in breast and prostate epithelial cells [124]. Previous studies by Powell et al. [46] showed that the ERβ selective agonist, liquiritigenin, which can bind to both ER subtypes, induces an ERα conformation that prefers heterodimerization with ERβ, as opposed to forming ERα homodimers. Therefore, we cannot exclude heterodimer formation as an explanation for the strong agonist effect of the *Cylopia* extracts in the transactivation model in MCF-7BUS cells.

Having evaluated the agonist and antagonist activity of *Cyclopia* extracts in a system where the ER subtypes were expressed separately and together, in a transactivation and a transrepression model, we increased the level of complexity by evaluating the effect of the extracts on MCF-7BUS cell proliferation, a system where the final cell phenotype is a product of not only the two ER subtypes but also of an integrated transactivation and transrepression system [39,98-100]. Although the *Cyclopia* extracts, like E_2_, induced cell proliferation it was with either a significantly lower potency (P104 and SM6Met) or lower efficacy (P104 and cup-of-tea) than E_2_. Furthermore, in the presence of E_2,_ all of the *Cyclopia* extracts displayed antagonistic properties. Similarly, the polyphenols also induced cell proliferation with either lower efficacies or potencies than E_2_ and some (genistein and enterodiol) also displayed antagonism. Previously, the agonist activity seen in the transactivation model in MCF-7BUS cells was ascribed to ERβ activation and this is probably translating into weak induction of MCF-7BUS cell proliferation. Furthermore, liquiritigenin, an ERβ selective agonist, although not able to induce significant MCF-7 cell growth in a mouse xenograft model [19,24], was able to induce proliferation of the ERα and ERβ positive [125] osteoblast-like murine MC3T3-E1 cells [126]. The antagonism of E_2_-induced cell proliferation by extracts of *Cyclopia* could be attributed to ERα antagonism (observed in the transactivation model in COS-1 cells), ER mediated repression of proliferation inducing genes (ER transrepression observed in MCF-7BUS transrepression model), ERβ-mediated transcription (observed in the transactivation model in COS-1 cells) of anti-proliferative and anti-apoptotic genes [39,127], or they might favor the formation of ERα/β heterodimers, which has been suggested to have growth inhibitory effects in breast epithelial cells [124]. 

Furthermore, we also evaluated the estrogenic and antiestrogenic properties of the *Cyclopia* extracts in an *in vivo* model, an immature rat uterotrophic assay. For the first time we show *in vivo* biological activity of the phytoestrogenic extracts of *Cyclopia*. SM6Met and cup-of-tea, unlike E_2_ and genistein, did not increase uterine weight and SM6Met, like the ER antagonist ICI 182,780, antagonized E_2_-induced uterine growth. The ERα subtype is the major subtype expressed in the uterus with very low levels of ERβ expressed [56,101]. Powell et al. [46] show that although ERβ homodimers and ERα/ERβ heterodimers are favored, genistein is capable of inducing ERα homodimers and activating ERα-induced transcription. Therefore, we can assume that the increase in uterine growth induced by genistein in the uterotrophic assay is a product of increased ERα homodimerization and hence, increased ERα mediated transcription. The ERβ selective agonists, liquiritigenin and ERB-041, in contrast, do not induce uterine growth [19,128]. Thus, the findings regarding ERβ selective agonists combined with the low levels of ERβ in the uterus excludes ERβ as the subtype eliciting the effect of *Cyclopia* extracts in the uterus. It is thus likely that the effect of *Cyclopia* extracts is due to ERα antagonism, as seen in the transactivation model in COS-1 cells, or that upon binding to the ER, the *Cyclopia* extracts induce a change in conformation that inhibits co-activator recruitment or activates co-repressor recruitment. The inability of the *Cyclopia* extracts to induce uterine growth, in contrast to MCF-7BUS cell proliferation, might also be attributed to either the differences in the concentration of co-regulators or the differences in co-regulator recruitment in the breast and uterus [129,130].

Having established ER agonist and/or antagonist activity of *Cyclopia* extracts, we look towards HPLC data, from the current and previous studies, to identify the polyphenol(s) responsible for the observed effects. The xanthones, mangiferin and isomangiferin, were identified in all *Cyclopia* extracts, but as mangiferin has no estrogenic potential, while isomangiferin has not previously been tested for estrogenicity [71], it is unlikely that the observed ER agonist/antagonist effects of *Cyclopia* can be ascribed to these polyphenols. However, mangiferin has been shown to inhibit the proliferation of breast cancer cells via ER independent mechanisms [131] and therefore, as mangiferin is present in all extracts at relatively high amounts it cannot be excluded as the polyphenol antagonizing E_2_-induced MCF-7BUS cell proliferation. Of the remaining polyphenols identified in the extracts the only aglycone present is the flavone, luteolin. *In vitro*, luteolin binds to both of the ER subtypes, is an ERα and ERβ agonist, induces MCF-7BUS cell proliferation, and antagonizes E_2_-induced MCF-7BUS cell proliferation [62,63,71,132-134]. Therefore, with regards to the *Cyclopia* extracts, the ERβ agonism observed in the transactivation model, the induction of MCF-7BUS cell proliferation, and the antagonism of E_2_-induced cell proliferation may be ascribed to the presence luteolin in the extracts, however, the observed ERα antagonism in the transactivation model cannot. Although luteolin is present in all extracts, the concentration is low. However, the 7-*O*-rutinoside of luteolin, scolymoside, is present in substantial amounts in all of the *C. subternata* extracts (presence was not evaluated in P104). This rutinoside of luteolin has not previously been tested for estrogenicity [71], however, as glycosides may be hydrolyzed by intestinal β-glucosidases [135,136], the bioavailability of the aglycone, luteolin, and hence phytoestogenicity of the extracts may increase upon hydrolysis of scolymoside. Furthermore, luteolin has been shown to have anti-tumor characteristics and can sensitize breast cancer cells to anti-tumor drugs such as tamoxifen [137] and therefore, the presence of luteolin, as well as scolymoside, in *Cylopia* extracts can be seen as positive regarding chemoprevention as well as breast cancer treatment. Generally, the glycosides of polyphenols either display reduced estrogenic activity compared to the aglycones or have not been evaluated for estrogenicity [71]. Thus, if the hydrolysis of glycosides present in the *Cyclopia* extracts is considered, it allows us to evaluate the phytoestrogenicity of the aglycones alongside their glycosides: apigenin (aglycone of vicenin-2), eriodictyol (eriocitrin), hesperitin (hesperidin), phloretin (phloretin-3,5-di-C-glucoside), hydroxyphloretin (3-hydroxyphloretin-3’,5’-di-*C*-hexoside), and iriflophenone (iriflophenone-2-*C*-β-glucoside and iriflophenone-di-*O*,*C*-hexoside). However, as β-glucosidases are produced by intestinal flora [138,139], consideration of glycoside metabolism will not help to identify the polyphenols responsible for *in vitro* results but may only be relevant for interpretation of *in vivo* results. For example, as luteolin and apigenin have been shown to significantly increase uterine weight, either in the presence or absence of estrogens [140,141], the effect elicited by *Cyclopia* extracts *in vivo* cannot be ascribed to luteolin, scolymoside, or vicenin-2. The effect of the other identified polyphenols has not been evaluated *in vivo* and therefore we cannot definitively attribute the *in vivo* effect of the *Cyclopia* extracts to any of these polyphenols. Of the glycosides, ericotrin and hesperidin have been tested for phytoestrogenicity *in vitro* [71]. However, hesperidin does not bind to the ER [62] or activate an ERE-containing promoter reporter construct [133]. Eriocitrin, however, has been shown to bind to only ERβ [62], but no work has been done to elucidate the estrogenic effect elicited by this polyphenol. For the first time we identified the dihydrochalcone, aspalathin, in *Cyclopia*. Aspalathin has not been tested for estrogenicity but has been shown to inhibit the proliferation of liver cells [142], however, due to the presence of unique drug metabolizing enzymes in the liver, the possibility of aspalathin metabolites eliciting this effect cannot be excluded nor can the results be extrapolated to breast cancer cells. The phytoestrogenicity of the remaining glycosides and aglycones, as well as protocatechuic acid, has not been tested [71]. In summary, none of the compounds identified in the *Cyclopia* extracts can account for the observed ERα antagonism, some (luteolin and eriocitrin) may explain the observed ERβ agonism and others (mangiferin and aspalathin) should not be excluded as possible effectors of ER-independent effects on proliferation. Therefore, thus far, we cannot with certainty ascribe the effects observed with *Cyclopia* extracts in this study to any of the individual constituents of our extracts. Although, further research regarding the polyphenol content, bioavailability, and estrogenic activity of our extracts is required to identify the compound causing the observed effects, we cannot exclude the possibility that a mixture of polyphenols is required to elicit the effects observed with *Cyclopia* extracts.

Physiologically, our results may be assessed both in terms of treatment of menopausal symptoms (hot flashes, osteoporosis, and increased inflammation [2-4,52,90-92]) and prevention of estrogen replacement associated side effects (breast cancer and uterine proliferation [5,6,52]). With regards to menopausal symptoms, the ERβ agonist MF101 [24], has been shown in clinical trials to reduce hot flashes and thus, the ERβ agonism of the *Cyclopia* extracts may be considered as a positive attribute. Furthermore, with regards to the postmenopausal surge in inflammatory disorders the fact that the *Cyclopia* extracts displayed agonism in the transrepression model in MCF-7BUS cells may also be considered as a positive attribute for the treatment of postmenopausal inflammatory disorders. With respect to the known roles of ER subtypes in breast cancer [15,22,37-43], the fact that extracts of *Cyclopia* antagonize ERα, while being ERβ agonists, may be beneficial. In addition, the extracts were able to antagonize the proliferation of breast cancer cells in the presence of E_2_ at lower concentrations than that required for breast cancer cell proliferation. Furthermore, not only do the *Cyclopia* extracts show potential as protectors against breast cancer development and inflammatory disorders, they also do this without promoting uterine growth, a negative SERM associated side effect [35,143].

Although *Cyclopia* extracts show potential to be developed as SERSMs, further work, which is ongoing, is needed to clarify their mechanism of action. This includes, but is not limited to, directly comparing the *Cyclopia* extracts with the known SERMs tamoxifen and raloxifene, investigating the effect of *Cyclopia* extracts on ER subtype levels, ER homo- or heterodimerization, induction or inhibition of co-regulator recruitment, and the modulation of cancer development and progression in a rat breast cancer model. In addition, further work is needed to identify the polyphenol(s) responsible for eliciting the observed effects and the possibility that distinct polyphenols present in *Cyclopia*, rather than an individual polyphenol, may be causing the observed ERα agonism and ERβ antagonism cannot be excluded. 

## Supporting Information

Figure S1 **Determination of ERE-containing promoter reporter construct concentration.** (A & B) COS-1 cells, transfected with equal amounts of (A) ERα and (B) ERβ, and (C) MCF-7BUS cells were transfected with increasing amounts of the ERE-containing promoter reporter construct (ERE.vit2.luc) and treated with either solvent or E_2_ to determine at which concentration of the ERE-containing promoter reporter construct the highest induction of E_2_ is observed. The dotted line through the bars represents the values for solvent control. Fold induction is indicated in boxes above the E_2_ columns. Average ± SEM is of one experiment done with three to four repeats.
(TIF)

Figure S2 **Determination of NFκB-containing promoter reporter construct concentration.** (A & B) COS-1 cells, transfected with equal amounts of (A) ERα and (B) ERβ, and (C) MCF-7BUS cells were transfected with increasing amounts of the NFκB-containing promoter reporter construct (p(IL6kB)350hu.IL6Pluc+) and treated with either solvent, PMA or PMA + E_2_ to determine at which concentration of the NFκB-containing promoter reporter construct the highest repression by E_2_ of PMA induction is observed. The dotted lines through the bars represent the values for either solvent control or 10ng/ml PMA. Percentage repression, where applicable, is indicated in boxes above the PMA + E_2_ columns. Average ± SEM is of one experiment done with three repeats.
(TIF)

Figure S3 **The effect of the SM6Met and cup-of-tea extracts on immature rat uterine growth.** Immature female wistar rats were treated with 2000, 200, and 20mg/kg body weight SM6Met and cup-of-tea for three consecutive days. Animals were sacrificed on day four, (A) uteri were photographed and (B) wet and (C) blotted uterine/final body weight was determined. One-way ANOVA with Dunnett’s post-test comparing all columns to solvent control (*, P<0.05; **, P<0.01; ***, P<0.001). The dotted line through the bars represents the values for solvent control. Average ± SEM is of at least eight animals/group.
(TIF)

Figure S4 **The effect of E_2_, genistein, extracts of *Cyclopia*, and ICI on body and liver weight.** Immature female wistar rats were treated for three consecutive days with 100µg/kg body weight (BW) E_2_, in the presence and absence of 2000mg/kg BW SM6Met or 10mg/kg BW ICI 182,780, 100mg/kg BW genistein, 2000, 200, or 20mg/kg BW SM6Met, 2000, 200, or 20mg/kg BW cup-of-tea, and 10mg/kg BW ICI 182,780 for three consecutive days. Animal were sacrificed on day four and changes in (A) body and (B) liver weights were determined. One-way ANOVA with Dunnett’s post-test comparing all columns to solvent control (*, P<0.05; **, P<0.01; ***, P<0.001). The dotted line through the bars represents the values for solvent control (A and B) and 100µg/kg BW E_2_ (A). Average ± SEM is of at least five animals/group.
(TIF)

Figure S5 **The effect of different concentration of the SM6Met and cup-of-tea extracts on the onset of vaginal opening.** Immature female wistar rats were treated for 30 consecutive days with the SM6Met and cup-of-tea extracts and the day of vaginal opening was determined. One-way ANOVA with Dunnett’s post-test comparing all columns to solvent control (*, P<0.05; **, P<0.01; ***, P<0.001). The dotted line through the bars represents the values for solvent control. Average ± SEM is of at least eight animals/group.
(TIF)

Table S1 **Summary of ER agonism and antagonism of *Cyclopia* extracts.**
(DOCX)
