# Correction: *Cyclopia* Extracts Act as ERα Antagonists and ERβ Agonists, *In Vitro* and *In Vivo*

**DOI:** 10.1371/annotation/3306cc63-2aab-42b1-98c1-7507705fff98

**Published:** 2013-12-20

**Authors:** Koch Visser, Morné Mortimer, Ann Louw

One of the polyphenols was named incorrectly throughout the manuscript. Aspalathin should be 3-hydroxyphloretin-3',5'-di-C-hexoside.

In addition, the last sentence of the "High-performance liquid chromatography (HPLC) analysis of C. subternata extracts" section of the Materials and Methods should read, "Also phloretin-3',5'-di-C-glucoside and 3-hydroxyphloretin-3',5'-di-C-hexoside were expressed in terms of nothofagin (phloretin-5'-C-glucoside) and aspalathin (3-hydroxyphloretin-5'-C-glucoside) equivalents, respectively."

In the 11th paragraph of the Discussion, the sentence "For the first time we identified the dihydrochalcone, aspalathin, in *Cyclopia*" is incorrect; it should read, "The dihydrochalcone, 3-hydroxyphloretin-3',5'-di-C-hexoside, has previously been identified in Cyclopia [75]." The following sentence should read, "3-Hydroxyphloretin-3',5'-di-C-hexoside has not been tested for estrogenicity, but aspalathin, a monoglucoside, has been shown to inhibit the proliferation of liver cells [142], however, due to the presence of unique drug metabolizing enzymes in the liver, the possibility of aspalathin metabolites eliciting this effect cannot be excluded nor can the results be extrapolated to breast cancer cells."

In Table 1, "Phloretin-3,5-di-C-glucoside" should instead read "Phloretin-3',5'-di-C-glucoside." 

