# Supplementary figures and images for: Cyclopia Extracts Act as ERα Antagonists and ERβ Agonists, In Vitro and In Vivo
Source: PLoS One. 2013 Nov 4;8(11):e79223. doi: 10.1371/journal.pone.0079223 (PMC3817056; doi:10.1371/journal.pone.0079223)

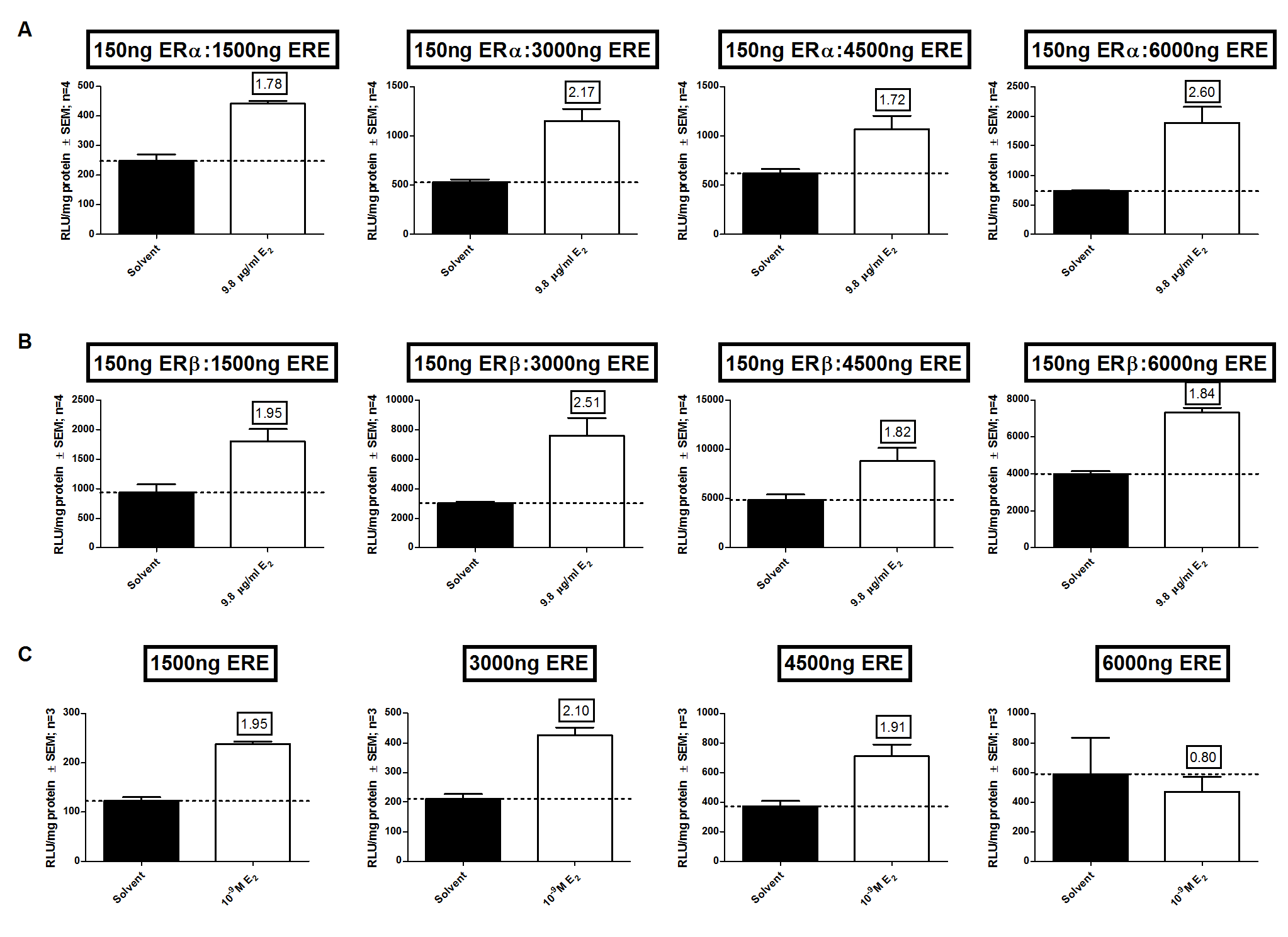

Supplement: Figure S1 — Determination of ERE-containing promoter reporter construct concentration. (A & B) COS-1 cells, transfected with equal amounts of (A) ERα and (B) ERβ, and (C) MCF-7BUS cells were transfected with increasing amounts of the ERE-containing promoter reporter construct (ERE.vit2.luc) and treated with either solvent or E2 to determine at which concentration of the ERE-containing promoter reporter construct the highest induction of E2 is observed. The dotted line through the bars represents the values for solvent control. Fold induction is indicated in boxes above the E2 columns. Average ± SEM is of one experiment done with three to four repeats. (TIF) [file pone.0079223.s001.tif]

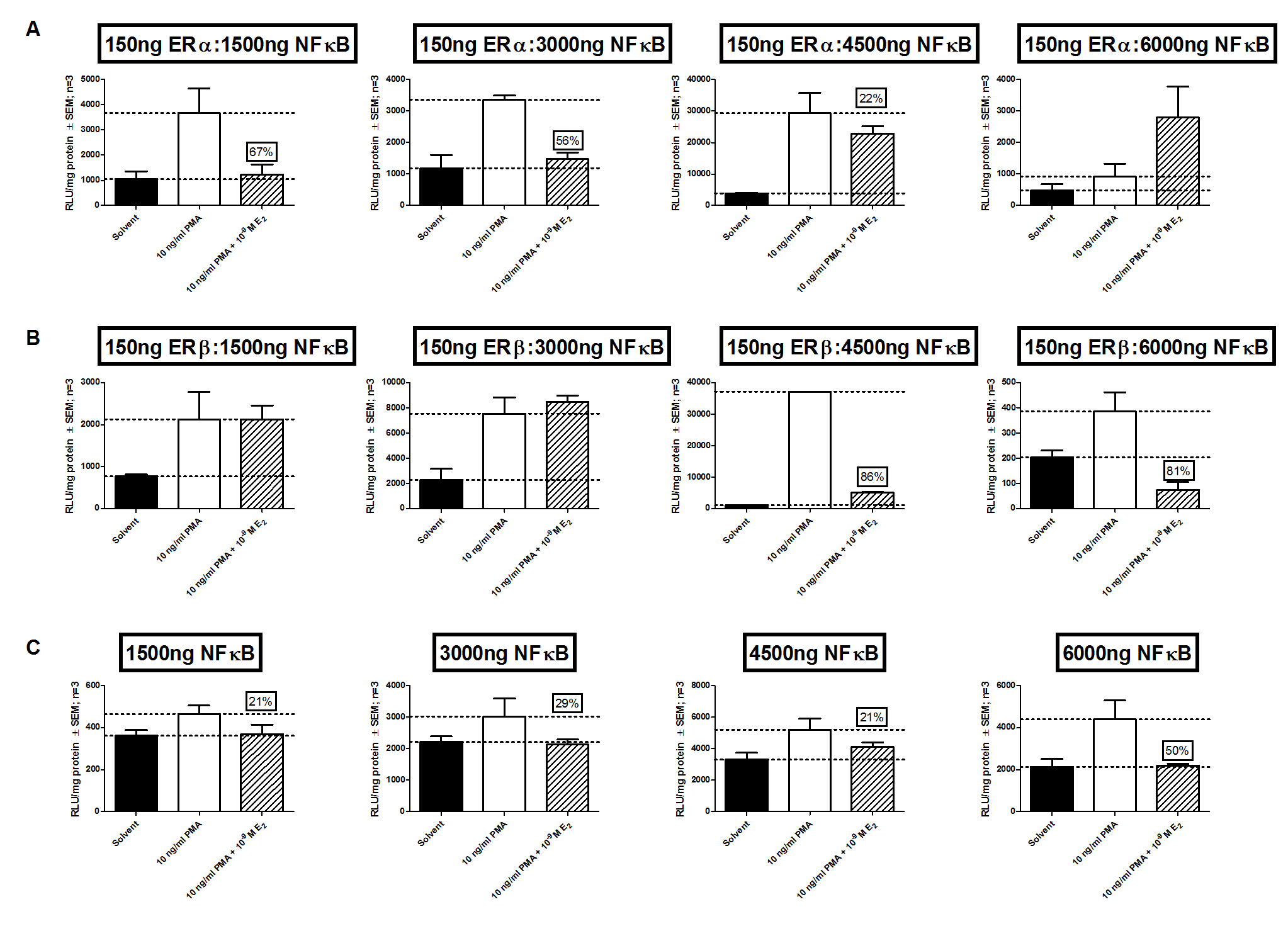

Supplement: Figure S2 — Determination of NFκB-containing promoter reporter construct concentration. (A & B) COS-1 cells, transfected with equal amounts of (A) ERα and (B) ERβ, and (C) MCF-7BUS cells were transfected with increasing amounts of the NFκB-containing promoter reporter construct (p(IL6kB)350hu.IL6Pluc+) and treated with either solvent, PMA or PMA + E2 to determine at which concentration of the NFκB-containing promoter reporter construct the highest repression by E2 of PMA induction is observed. The dotted lines through the bars represent the values for either solvent control or 10ng/ml PMA. Percentage repression, where applicable, is indicated in boxes above the PMA + E2 columns. Average ± SEM is of one experiment done with three repeats. (TIF) [file pone.0079223.s002.tif]

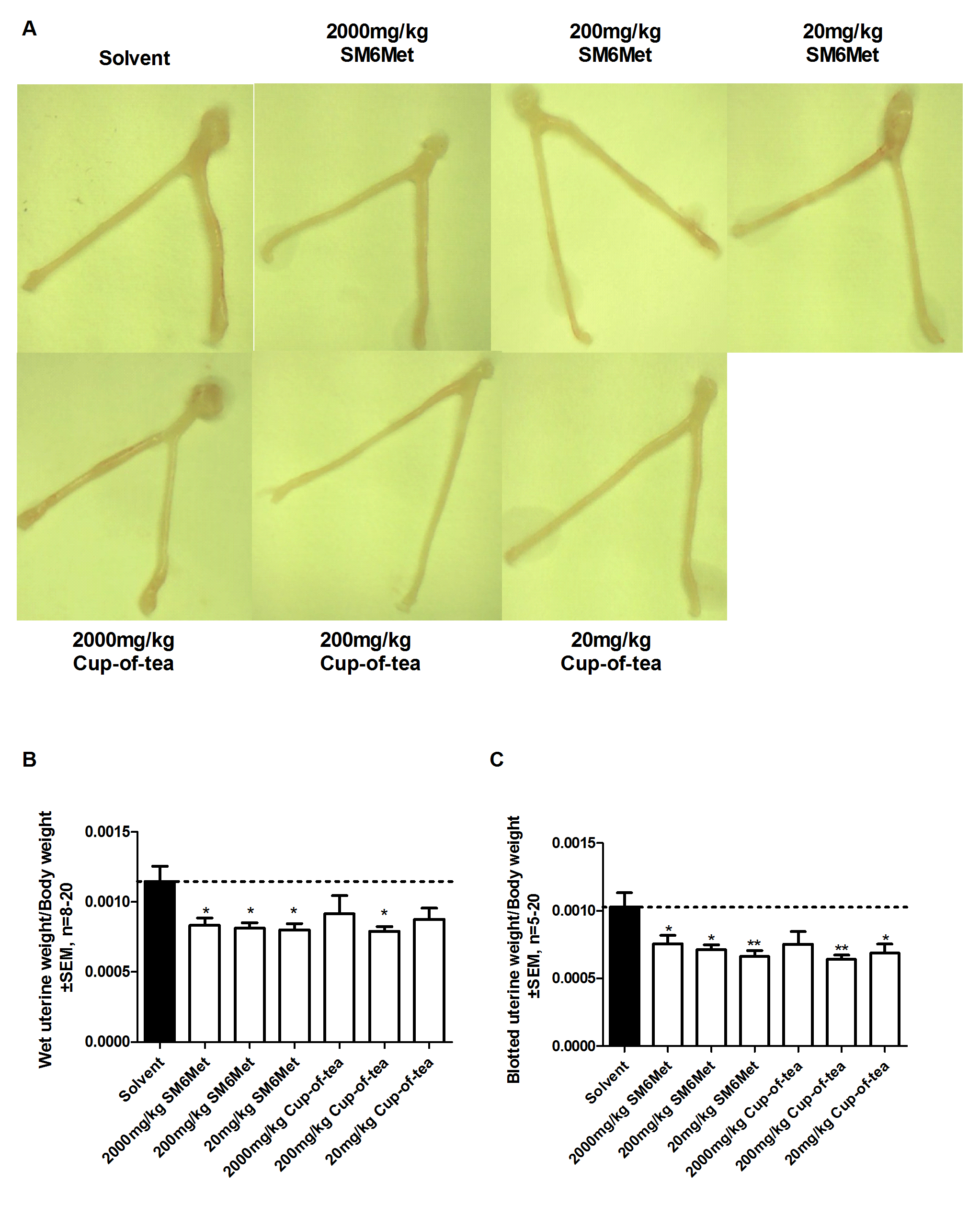

Supplement: Figure S3 — The effect of the SM6Met and cup-of-tea extracts on immature rat uterine growth. Immature female wistar rats were treated with 2000, 200, and 20mg/kg body weight SM6Met and cup-of-tea for three consecutive days. Animals were sacrificed on day four, (A) uteri were photographed and (B) wet and (C) blotted uterine/final body weight was determined. One-way ANOVA with Dunnett’s post-test comparing all columns to solvent control (*, P<0.05; **, P<0.01; ***, P<0.001). The dotted line through the bars represents the values for solvent control. Average ± SEM is of at least eight animals/group. (TIF) [file pone.0079223.s003.tif]

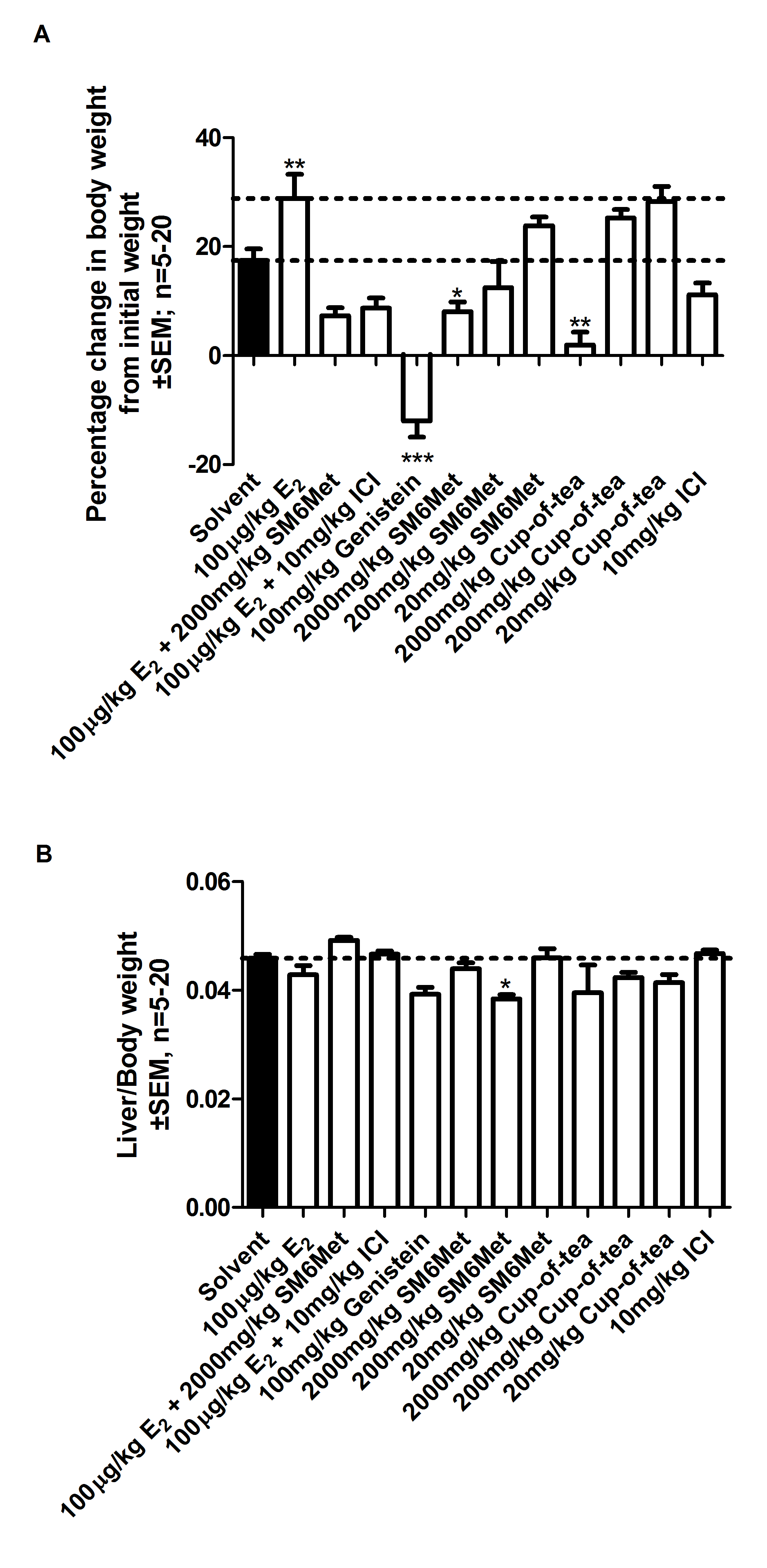

Supplement: Figure S4 — The effect of E2, genistein, extracts of Cyclopia, and ICI on body and liver weight. Immature female wistar rats were treated for three consecutive days with 100µg/kg body weight (BW) E2, in the presence and absence of 2000mg/kg BW SM6Met or 10mg/kg BW ICI 182,780, 100mg/kg BW genistein, 2000, 200, or 20mg/kg BW SM6Met, 2000, 200, or 20mg/kg BW cup-of-tea, and 10mg/kg BW ICI 182,780 for three consecutive days. Animal were sacrificed on day four and changes in (A) body and (B) liver weights were determined. One-way ANOVA with Dunnett’s post-test comparing all columns to solvent control (*, P<0.05; **, P<0.01; ***, P<0.001). The dotted line through the bars represents the values for solvent control (A and B) and 100µg/kg BW E2 (A). Average ± SEM is of at least five animals/group. (TIF) [file pone.0079223.s004.tif]

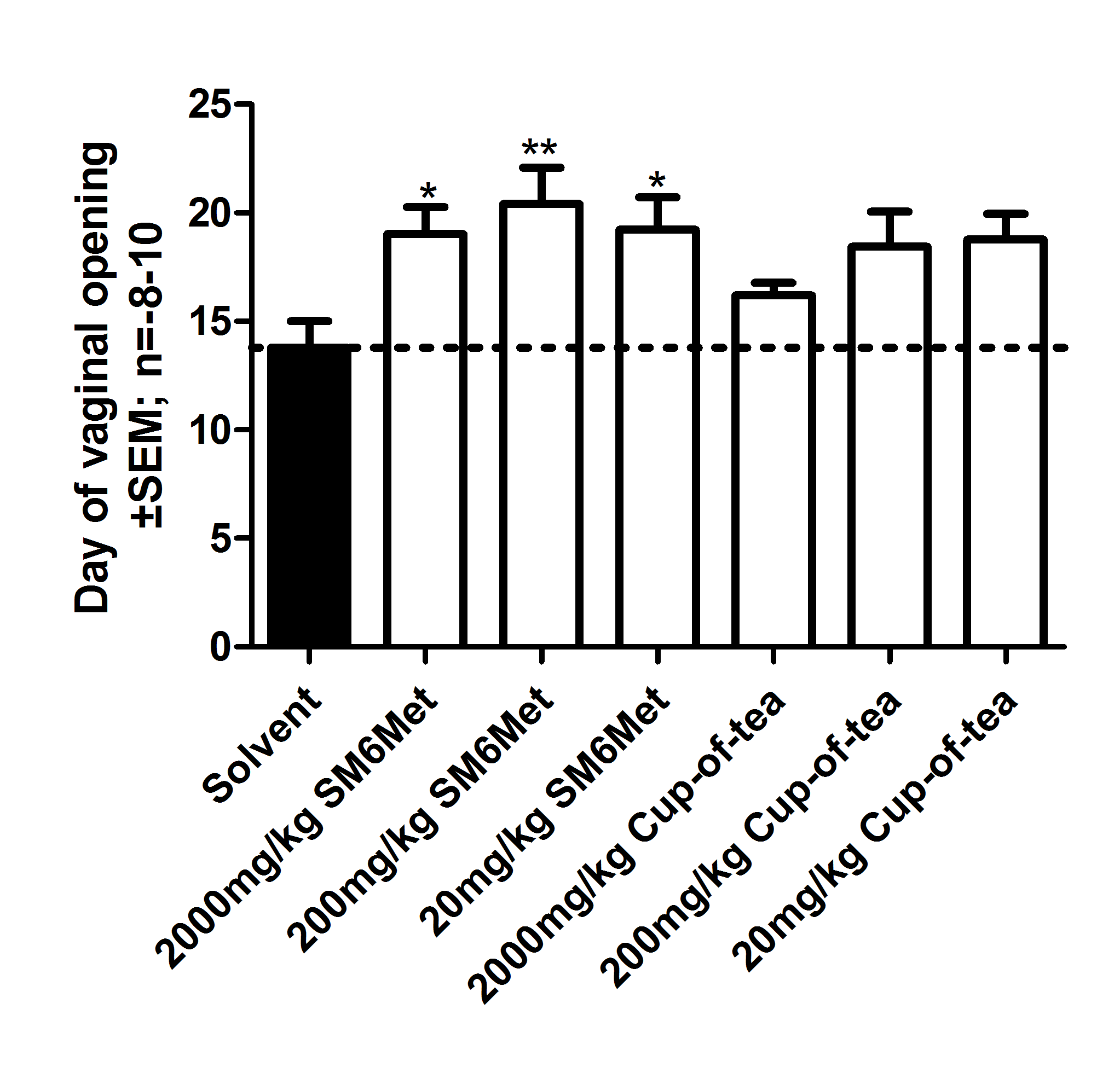

Supplement: Figure S5 — The effect of different concentration of the SM6Met and cup-of-tea extracts on the onset of vaginal opening. Immature female wistar rats were treated for 30 consecutive days with the SM6Met and cup-of-tea extracts and the day of vaginal opening was determined. One-way ANOVA with Dunnett’s post-test comparing all columns to solvent control (*, P<0.05; **, P<0.01; ***, P<0.001). The dotted line through the bars represents the values for solvent control. Average ± SEM is of at least eight animals/group. (TIF) [file pone.0079223.s005.tif]
