# Supplementary material for: Cyclopia Extracts Act as ERα Antagonists and ERβ Agonists, In Vitro and In Vivo
Source: PLoS One. 2013 Nov 4;8(11):e79223. doi: 10.1371/journal.pone.0079223 (PMC3817056; doi:10.1371/journal.pone.0079223)
Supplement: Table S1 — Summary of ER agonism and antagonism of Cyclopia extracts. (DOCX) [file pone.0079223.s006.docx]

| **Extract** | **Type of extract** | **Species** | **Test model** | **Agonist*^a^*** | **Antagonist*^b^*** | **Reference** |
| --- | --- | --- | --- | --- | --- | --- |
| **P104** | Methanol | *C. genistodes* | **Transactivation**: COS-1 cells + hERα/hERβ & ERE-promoter reporter construct. | ERβ | nt*^c^* | [1] |
|  |  |  |  | ERβ | ERα | Fig. 1 |
|  |  |  | **Transactivation**: MCF-7BUS cells + ERE-promoter reporter construct. | ER | nt | Fig. 2 |
|  |  |  | **Transrepression**: COS-1 cells + hERα/hERβ & NFκB-promoter reporter construct. | ERα & ERβ | -*^d^* | Fig. 3 |
|  |  |  | **Transrepression**: MCF-7BUS cells + NFκB-promoter reporter construct. | ER | - | Fig. 4 |
|  |  |  | **Cell proliferation**: MCF-7BUS cells | ER | ER | [1] |
|  |  |  |  | ER | ER | Figs. 5, 6 |
| **SM6Met** | Methanol | *C. subternata* | **Transactivation**: ERα and ERβ expressing T47D-KBluc cells stably transfected with ERE-promoter reporter construct. | ER | nt | [2] |
|  |  |  | **Transactivation**: COS-1 + hERα/hERβ & ERE-promoter reporter construct. | ERβ | ERα | Fig. 1 |
|  |  |  | **Transactivation**: MCF-7BUS + ERE-promoter reporter construct. | ER | - | Fig. 2 |
|  |  |  | **Transrepression**: COS-1 cells + hERα/hERβ & NFκB-promoter reporter construct. | - | ERα*^e^* & ERβ | Fig. 3 |
|  |  |  | **Transrepression**: MCF-7BUS cells + NFκB-promoter reporter construct. | ER | - | Fig. 4 |
|  |  |  | **Cell proliferation**: MCF-7BUS cells | ER | nt | [2] |
|  |  |  |  | ER | ER | Figs. 5, 6 |
|  |  |  | **Immature rat uterotrophic assay** | - | ERα | Fig. 7 |
|  |  |  | **Vaginal opening** | - | ERα*^f^* | Fig. 8 |
| **Cup-of-tea** | Water | *C. subternata* | **Transactivation**: COS-1 cells + hERα/hERβ & ERE-promoter reporter construct. | - | ERα | Fig. 1 |
|  |  |  | **Transactivation**: MCF-7BUS cells + ERE-promoter reporter construct. | ER | - | Fig. 2 |
|  |  |  | **Transrepression**: COS-1 cells + hERα/hERβ & NFκB-promoter reporter construct. | - | ERβ & weak ERα | Fig. 3 |
|  |  |  | **Transrepression**: MCF-7BUS + NFκB-promoter reporter construct. | ER | ER | Fig. 4 |
|  |  |  | **Cell proliferation**: MCF-7BUS cells | ER | nt | [2] |
|  |  |  |  | Weak ER | ER | Figs. 5,6 |
|  |  |  | **Immature rat uterotrophic assay** | - | ERα | Sfig. 1 |
|  |  |  | **Vaginal opening** | - | Weak ERα | Sfig. 3 |

*^a^*Tested in the absence of E_2_.

*^b^*Tested in the presence of 10^-9^ M E_2_.

*^c^*nt = not tested

*^d^*- = no effect

*^e^*Tested in the absence of exogenous estrogens.

*^f^*Tested in the presence of endogenous estrogens.

1. Verhoog NJ, Joubert E, Louw A. (2007) Evaluation of the phytoestrogenic activity of *Cyclopia genistoides* (honeybush) methanol extracts and relevant polyphenols. J Agric Food Chem 55: 4371-4381.

2. Mfenyana C, DeBeer D, Joubert E, Louw A. (2008) Selective extraction of *Cyclopia* for enhanced *in vitro* phytoestrogenicity and benchmarking against commercial phytoestrogen extracts. J Steroid Biochem Mol Biol 112: 74-86
